# Impact of *ABCB1* and *CYP2B6* Genetic Polymorphisms on Methadone Metabolism, Dose and Treatment Response in Patients with Opioid Addiction: A Systematic Review and Meta-Analysis

**DOI:** 10.1371/journal.pone.0086114

**Published:** 2014-01-29

**Authors:** Brittany B. Dennis, Monica Bawor, Lehana Thabane, Zahra Sohani, Zainab Samaan

**Affiliations:** 1 Department of Clinical Epidemiology and Biostatistics, McMaster University, Hamilton, Ontario, Canada; 2 Biostatistics Unit, Father Sean O'Sullivan Research Centre, St Joseph's Healthcare, Hamilton, Ontario, Canada; 3 Population Genomics Program, McMaster University, Hamilton, Ontario, Canada; 4 McMaster Integrative Neuroscience Discovery & Study (MiNDS) Program, McMaster University, Hamilton, Ontario, Canada; 5 Departments of Pediatrics and Anesthesia, McMaster University, Hamilton, Ontario, Canada; 6 Population Health Research Institute, Hamilton Health Sciences, Hamilton, Ontario, Canada; 7 Department of Psychiatry and Behavioral Neurosciences, McMaster University, Hamilton, Ontario, Canada; Yale University, United States of America

## Abstract

**Background:**

Genetic variability may influence methadone metabolism, dose requirements, and risk of relapse.

**Objectives:**

To determine whether the CYP2B6*6 or *ABCB1* (rs1045642) polymorphisms are associated with variation in methadone response (plasma concentration, dose, or response to treatment).

**Methods:**

Two independent reviewers searched Medline, EMBASE, CINAHL, PsycINFO, and Web of Science databases. We included studies that reported methadone plasma concentration, methadone response, or methadone dose in relation to the CYP2B6*6 or *ABCB1* polymorphisms.

**Results:**

We screened 182 articles and extracted 7 articles for inclusion in the meta-analysis. Considerable agreement was observed between the two independent raters on the title (kappa, 0.82), abstract (kappa, 0.43), and full text screening (kappa, 0.43). Trough (R) methadone plasma concentration was significantly higher in CYP2B6*6 homozygous carriers when compared to non-carriers (standardized mean difference [SMD] = 0.53, 95% confidence interval [CI], 0.05–1.00, p = 0.03) with minimal heterogeneity (I^2^ = 0%). Similarly, trough (S) methadone plasma concentration was higher in homozygous carriers of the *6 haplotype when compared to non-carriers, (SMD = 1.44, 95% CI 0.27–2.61, p = 0.02) however significant heterogeneity was observed (I^2^ = 69%). Carriers of the CYP2B6*6 haplotype were not found to be significantly different from non-carriers with respect to dose or response to treatment. We found no significant association between the *ABCB1* polymorphism and the trough (R), (S) plasma concentrations, methadone dose, or methadone response.

**Conclusion:**

Although the number of studies included and sample size were modest, this is the first meta analysis to show participants homozygous for the CYP2B6*6 genotype have higher trough (R) and (S) methadone plasma concentrations, suggesting that methadone metabolism is significantly slower in *6 homozygous carriers.

## Introduction

Methadone maintenance therapy (MMT) is a substitute opioid therapy (SOT) used to treat opioid withdrawal symptoms. SOT is a harm reduction approach aimed to treat the symptoms of opioid withdrawal in a controlled environment. Currently methadone is the most common and efficaciously used SOT in the treatment of opioid addiction [1], [2]. Methadone has been associated with a reduction in continued opioid abuse, mortality, criminal acts, and infectious disease [2]–[7]. Methadone is formed by a racemic mixture of (R) and (S) enantiomers, where the (R) enantiomer accounts for the complete opioid effect felt by the patients [8].

Continued opioid abuse is one of the most common risk factors for mortality among patients in MMT, often because of the high risk for overdose when taking methadone in concurrence with other opioids [9]. The risk of mortality is among one of the serious problems MMT patients face. Fifteen to twenty percent of the patients most negatively affected, such as high-risk IV drug users with concurrent infectious diseases, have a poor response to MMT [10], [11]. Such patients have limited retention in treatment or they continue abusing illicit substances while in treatment [10], [12], [13]. A patient's response to opioid addiction treatment is subject to high inter-individual variability, arguably due to the numerous environmental, social, and genetic influences on MMT response. Genetic predisposition as a risk factor for opioid addiction has been reported in the literature, accounting for as high as 70% of the risk, thus implicating addiction as a largely heritable disorder [14]. Genetic variants among MMT patients can influence mortality [15], patient satisfaction with methadone treatment [16], dose requirements [16]–[24], methadone metabolism [8], [17], [18], [20], [24], withdrawal symptoms [23], [25], and risk of relapse [18], [20], [26]–[29]. Genetic variability is now being cited as a large contributor to the variability in MMT patient response [30]. If genetic factors are associated with low methadone plasma concentrations, high methadone dose, or drug use behaviors, we may be able to adequately identify patients who are at risk for poor MMT response, and ultimately tailor treatment to improve patient health outcomes and optimal dosing strategies.

While meta-analyses have been completed to examine the genetic determinants of opioid addiction [31], [32], no systematic reviews or meta-analyses to date have been conducted to investigate the association between genetic polymorphisms and methadone dose, metabolism, or treatment response. We therefore conducted a rigorous systematic review of the available literature to determine firstly, which genes were most investigated and associated with a range of methadone outcomes, including but not limited to: methadone response, trough (R) and (S) methadone plasma concentration, and methadone dosing. Criteria were established a priori when determining the most important single nucleotide polymorphisms (SNPs) or haplotypes to evaluate in this systematic review. The SNP or haplotype had to be evaluated in more than four studies to allow for subgroup and sensitivity analyses when possible and had to be investigated within the context of a MMT patient population. The authors used a sensitive search strategy in OVID MEDLINE when originally determining the genetic polymorphisms that have been studied most. This search included the terms, “Methadone,” AND “Opioid Addiction,” AND “Genes,” OR “Genetic Polymorphisms,” OR “Single nucleotide polymorphisms,” which led to the location of the genes and SNPs analyzed in this review. However, this original search was preliminary and not done in duplicate. The purpose of this search was to help us develop the research question by locating articles on the genetic determinants of methadone response. After applying these criteria to a preliminary search of the genetic determinants of MMT response, we were able to locate two polymorphisms of interest, these being the ATP-binding cassette subfamily B member 1 (*ABCB1*) SNP (rs1045642) and the Cytochrome p450 2B6 *6 haplotype SNPs. The *ABCB1* SNP and *CYP2B6* haplotype were the most widely reviewed genetic determinants within the methadone literature. See Table S1 for a list of the SNPs reviewed during the process of SNP selection, which was constructed to outline all the SNPs reviewed in the preliminary search, and all available studies were cited within this chart. Before explicitly addressing the objectives of this systematic review, we will first provide background information on the MMT treatment regime and the genes of interest for this review.

The most consistent gene that is implicated in MMT drug response is the ATP-binding cassette, sub family B, member 1 (*ABCB1*) gene, which is the primary focus of this study. The *ABCB1* gene is located on chromosome 7, and is responsible for encoding the efflux drug transporter P glycoprotein [21]. Literature has reported 38 known SNPs on the coding region that have varying allelic frequencies among different populations [21]. The most common variant in the coding sequence is 3435C>T (rs1045642), located on exon 26, which has been previously examined in substance abuse populations [21], [33], [34]. To date, few studies have investigated the association between the rs1045642T allele and methadone related outcomes, such as methadone dose, metabolism, and response. There is evidence supporting the genetic variability of this SNP and its effect on methadone metabolism, with the homozygous TT carriers requiring a higher methadone dose [30], [33]. A study by Levran (2008) used the rs1045642 SNP as part of a haplotype analysis and found similar findings, where the homozygous T carriers were found to have higher methadone doses [21].

In addition, the impact of rs1045642 has been explored in methadone metabolism and pharmacokinetics. Crettol et al. (2006) studied methadone metabolism by observing trough (R,S) methadone plasma levels among various populations and found that individuals possessing the rs1045642TT genotype had lower methadone plasma levels, indicating faster methadone metabolism [17]. However, other studies have reported conflicting results; both Fonseca et al. (2011) and Lotsch et al. (2006) found no genotypic differences in methadone plasma concentrations [20], [35], [36]. Lastly, studies examining the impact of *ABCB1* polymorphisms on response to methadone maintenance treatment also report opposing findings [17], [20]. Overall, the literature suggests that *ABCB1* polymorphisms may impact methadone dose requirements, plasma concentration, and response to treatment, however conclusions are inconsistent and require further investigation.

Cytochrome p450 2B6 (*CYP2B6*) has also been commonly studied in opioid dependence and drug response, and as such will be another focus of this study. *CYP2B6* is located on chromosome 19 and is responsible for coding metabolic enzymes of the Cytochrome P450 family [20], [35]. There is a large inter-individual variability in the mRNA expression and activity of *CYP2B6*, which can in part be explained by genetic polymorphisms [18]. Current evidence suggests that the commonly reported CYP2B6*6 haplotype, a combination of the *9 (rs3745274, c516G >T;Q172H), and *4 (rs2279343, c785A>G; K262R) SNPs, influences opioid addiction and methadone related outcomes [17], [20]. Studies looking at this specific variant have shown that it affects methadone metabolism, demonstrating higher plasma levels of S-methadone in Caucasian populations [18]. These haplotype carriers also showed significantly higher trough (S)methadone plasma levels and a trend towards higher (R)methadone plasma levels in a study by Crettol et al. (2006) [17]. With regards to response to treatment, varying conclusions have been found among studies, with some supporting the role of the *6 haplotype in MMT response and others finding no significant associations [17], [20]. Studies investigating the role of the *CYP2B6* haplotype in methadone dose requirements have also found inconsistent results, making it difficult to draw a direct conclusion for this particular outcome. However, it is suggested that carriers of the *6 variant may require lower doses than non-carriers [17], [20]. In addition, studies also report associations between the minor allele (T) frequencies of the rs3745274 SNP in the *CYP2B6* haplotype variant and methadone clearance, plasma levels, and dose [24]. Contradictory studies are also present in the literature, showing no significant metabolic differences between genotype frequencies and methadone related variables [20]. Data regarding *CYP2B6* and its role in drug response are inconsistent; therefore the aim of this review is to systematically combine these results to reach a general conclusion.

This current study aims to perform a systematic review to investigate the association between *CYP2B6* and *ABCB1* genetic polymorphisms and methadone maintenance therapy (MMT) patient response. For the purpose of this systematic review, individual patient response to MMT as an outcome will be analyzed separately for: methadone dose, methadone blood level, and methadone response as defined by the absence of illicit opioids use. Please refer to Table S2 for an explicit outline of the individual research questions. We constructed Table 1 in an effort to adequately understand how methadone outcomes are currently defined, reported, and analyzed in the available literature. The objectives of the systematic review are to:

**Table 1:** Methadone Maintenance Therapy Outcome Definitions, Measurements, and Statistical Measurement of Association in Genetic Studies.

MMT Response Outcome	Definition	Type of Variable	Measurement of Variable (units)	Statistical Estimates and Measurement of Association of this Outcome in Genetic Studies on MMT Patients	Studies
Methadone Dose	Average and maximum daily methadone dose during the first year of treatment	Continuous variable	Self report and chart review (dose in mg/day)	OR, Independent T-Test, Linear Regression Analysis, Proportional odds, Mann-Whitney U-test (for 2 groups), Kruskal-Wallis test (for **>**2 groups), linear regression	[17], [18], [21], [22], [30], [33], [40], [41]
Methadone Metabolism	Looking at methadone plasma levels in MMT patients, measuring the steady-state trough (R)- (i.e., the active enantiomer), (S)-, and (R,S)	Continuous variable	Steady-state trough and peak (R)-, (S)-, and (R,S)-plasma levels and peak-to-trough plasma level ratios (ng . kg/mL)	ANOVA, Mann-Whitney U-test (for 2 groups), Kruskal-Wallis test (for **>**2 groups)	[8], [17], [18], [20], [24]
Methadone Adherence	Abstinence from opioid for a period generally >2 months.	Binary Variable (responders or non-responders)	Self Reporting and Urine Toxicology Screening	Mann-Whitney U test (for 2 groups) and the Kruskal-Wallis test (for **>**2 groups), chi-square, OR	[8], [17]

1. Determine whether or not there is a genetic predisposition among MMT patients for any one of the aforementioned outcomes related to MMT response;
2. When appropriate, combine the results of the studies found in this systematic review in a meta analysis in an effort to estimate a mean difference, relative risk, or odds ratio that reflect the results of multiple studies in a summary estimate;
3. Evaluate where the gaps in the current literature are in an effort to determine the important questions that need be answered in future research;
4. Using the results of this systematic review and meta-analysis to provide unbiased estimates that will in effect improve the current understanding of MMT treatment practices.

## Methods

A specific protocol was designed for this systematic review and is available upon request. The protocol was registered with PROSPERO in December 2012 at http://www.crd.york.ac.uk/prospero/.

The electronic databases Medline, EMBASE, CINAHL, PsycINFO, and Web of Science were reviewed using a comprehensive search strategy. Please refer to Table 2 for an example of our MEDLINE search, and Table S3 for the full search strategy. It is important to note that the OVID Medline database encompasses PubMed within the search. Separate key terms were evaluated for use in the search strategy in an effort to adequately locate the necessary articles pertaining to the aforementioned research questions outlined in section 3.1. A McMaster University Faculty of Health Science librarian was consulted during the selection of databases and during the creation of the search strategy. Two independent reviewers (Bawor, M and Dennis, B) completed the title, abstract, and full text screening in duplicate, in addition to an individual search of the bibliographies to locate additional literature from all studies that passed the abstract screening stage. No language restrictions were put on this systematic review. The search has been restricted to human studies. In addition, only published literature were allowed into the systematic review for full data abstraction. Authors were contacted during the data extraction process to inform them about the review and request additional results related to their published works when needed during data abstraction.

**Table 2:** MEDLINE Search Strategy for Systematic Review and Meta–Analysis on the Genetic Determinants of Methadone Maintenance Therapy Response.

Medline Search Strategy = 54	1. methadone/bl, me, pk, th [Blood, Metabolism, Pharmacokinetics, Therapy]
	2. limit 1 to humans
	3. methadone .mp.
	4. opioid substitution treatment.mp. or Opiate Substitution Treatment/
	5. limit 4 to humans
	6. substance Related Disorders/bl, dt, ge, me [Blood, Drug Therapy, Genetics, metabolism]
	7. limit 6 to humans
	8. genetic polymorphism.mp. or Polymorphism, Genetic/
	9. limit 8 to humans
	10. single nucleotide polymorphism.mp. or Polymorphism, Single Nucleotide/
	11. limit 10 to humans
	12. polymorphism, Genetic/or Polymorphism, Single Nucleotide/or Genetic Variation/or genetic variant.mp. or Phenotype/
	13. limit 12 to humans
	14. Genes, MDR/or Polymorphism, Genetic/or ABCB1.mp. or Polymorphism, Single Nucleotide/
	15. limit 14 to humans
	16. cytochrome P450 Enzyme System/or CYP*.mp.
	17. limit 16 to humans
	18. methadone .tw.
	19. limit 18 to humans
	20. 2 OR 5 OR 7
	21. 3 AND 20
	22. 9 OR 11 OR 13
	23. 15 OR 17
	24. 22 AND 23
	25. 18 AND 24

**Figure 1:**
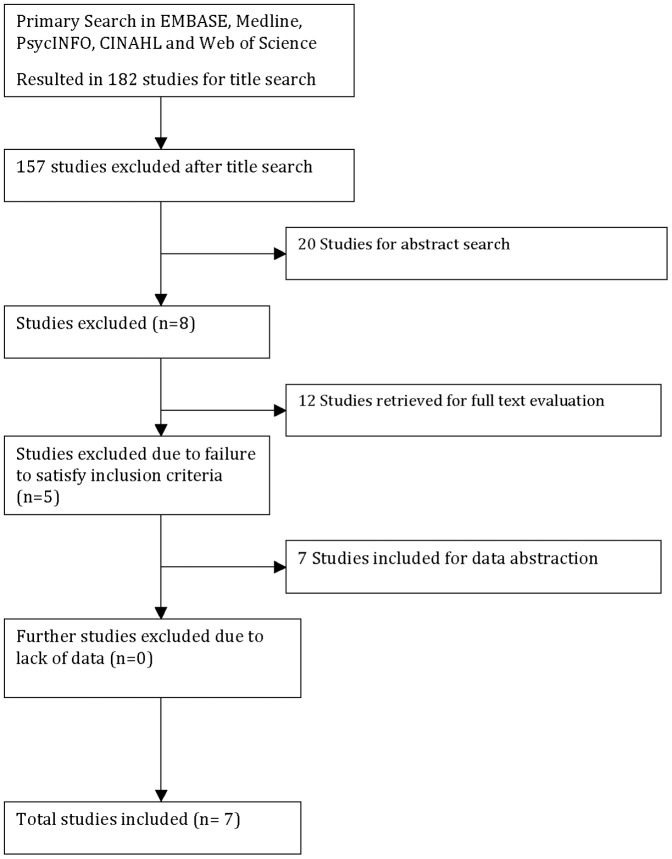
Methods for Extraction and Evaluation of Pertinent Studies.

The literature search encompassed an initial title search, title screening, abstract screening, and full text extraction. Both independent reviewers were responsible for completing: 1) the title search using the aforementioned search strategy, 2) the title and abstract screening with the use of pre determined inclusion criteria, 3) determining the eligibility of articles, and 4) the full data abstraction on eligible articles. Any disagreements that arose during the literature search and screening process were resolved by discussion, however a third party (Samaan, Z) was brought in to resolve disagreements when discussion could not. Both reviewers used the inclusion criteria determined a priori as the guide for determining the eligibility of an article. The kappa statistic was used to calculate level of agreement between independent raters [37]. In accordance with the meta analysis of observational studies in epidemiology (MOOSE) reporting guidelines, both a flow diagram of article selection (Figure 1) and detailed table of selected studies (Tables 3 – 5) are included in the systematic review [38].

**Table 3:** Summary of Findings Table for Individual Studies Selected for Full–Text Extraction.

First Author Last Name, Year of Publication	Journal of Publication	Title of Publication	(N)	% Male	Mean Age (SD)	Genes Assesse d	Ethnicity	Outcomes Assessed	Outcome Measures
Coller, 2006 [33]	Clinical Pharmacology and Therapeutics	*ABCB1* genetic variability and methadone dose requirements in opioid­ dependent individuals	60	68.3	32.1 (7.9)	*ABCB1*	92% Caucasian	*ABCB1* genetic variability on methadone dose requirements	Methadone dose measured as mean of doses (for subjects in treatment >2 months) or dose on day 40 (subjects in treatment <2 months)
Crettol, 2005 [18]	Clinical Pharmacology and Therapeutics	Methadone enantiomer plasma levels *CYP2B6*, *CYP2C19*, and *CYP2C9* genotypes, and response to treatment	209	76.6	36 (8)	*CYP2B6*, *CYP2C19* , and *CYP2C9*	95% Caucasian	Methadone pharmacokinetic s and response to MMT treatment	Review of trough and peak methadone R, S, and RS- plasma concentrations And methadone response measured by self declaration of opioid/cocaine abstinence and confirmed through urine toxicology screening
Crettol, 2006 [17]	Clinical Pharmacology and Therapeutics	*ABCB1* and cytochrome P450 genotypes and phenotypes: influence on methadone plasma levels and response to treatment	245	75.5	36 (8)	*ABCB1*, *CYP2B6*, *CYP2D6*, *CYP3A5*, *CYP1A2*, *CYP2C9*, *CYP2C19*	95% Caucasian	Methadone kinetics (methadone plasma levels) and response to treatment	Review of trough and peak methadone R, S, and RS­ plasma concentrations And methadone response measured by self declaration of opioid/cocaine abstinence and confirmed through
									urine toxicology screening
Fonseca, 2011 [20]	PLoS ONE	Contribution of Cytochrome P450 and *ABCB1* Genetic Variability on Methadone Pharmacokinetics, Dose Requirements, and Response	105	71	38 (8)	*ABCB1*, *CYP2B6*, *CYP2D6*, *CYP3A5*, *CYP2C9*, *CYP2C19*	Caucasian	Response to treatment and methadone pharmacokinetic s.	Response was measured through the use of urine toxicology screening. Pharmacokinetics was determined by reviewing the participants trough methadone R, S, and RS- plasma concentrations
Hung, 2011 [30]	Pharmacogenomics	Impact of genetic polymorphism in *ABCB1*, *CYP2B6*, *OPRM1*, *ANKK1*, and *DRD2* genes on methadone therapy in Han Chinese patients	321	78.8	36.5 (18.7)	*ABCB1*, *CYP2B6*, *OPRM1*, *ANKK1*, *DRD2*	Han Chinese	The genetic determinants of methadone dose requirements, through the comparison of dose among risk allele carriers to non risk allele carriers	methadone dose divided into 3 groups: <55 mg/day, 55–99 mg/day, 100–150 mg/day. Patients were genotyped and the mean dose among these genotypes were compared.
Levran, 2008 [21]	Human Molecular Genetics	*ABCB1* (MDR1) genetic variants are associated with methadone doses required for effective treatment of heroin dependence	98	65.3	45	*ABCB1*	Jewish Ancestry	The genetic determinants of methadone dose.	Methadone dose divided into 2 groups: 30–150 mg/day and 151–280 mg/day
Uehlinger, 2007 [48]	Journal of Clinical Psychopharmacolog y	Increased (R)­ Methadone Plasma Concentrations by Quetiapine in cytochrome P450s and *ABCB1* genotyped patients	14	78.5	34	*ABCB1*, *CYP2B6*, *CYP2D6*	Caucasian	The effect of quetiapine on methadone metabolism	Trough plasma concentrations of R, S, and RS­ methadone (before and after quetiapine)

**Table 4:** Summary of Findings Table for Outcome Data in *ABCB1* Studies.

First Author Last Name, Year of Publication	SNPs	Minor Allele Frequency	Outcome(s) Assessed	Statistical Measures of Association	Methadone Dose (mg/d) by Genotype	Results/Conclusion for Methadone Plasma Concentrations	Response to Treatment
Coller, 2006 [33]	rs1045642	28.3% (T)	Determine the frequency of *ABCB1* haplotypes formed by the A61G, G1199A, C1236T, G2677T, and C3435T SNPs. Investigate the relationships between *ABCB1* haplotypes and P- glycoprotein function as assessed by methadone dose requirements among opioid-dependent patients.	Odds ratio and Fisher exact test	Not reported.	No statistically significant differences found (p = >0.05) when comparing methadone dose genotype profiles for the *ABCB1* rs1045642 single nucleotide polymorphism	n/a
Crettol, 2006 [17]	rs1045642	24.5% (T)	Methadone kinetics (methadone plasma levels) and response to treatment (comparison of high dose and low dose responders to high dose non­ responders).	Log transformed data used for comparison between genotype and phenotype groups with independent t- test/1 way ANOVA for linear regression	Not reported.	*ABCB1* carriers of rs1045642 risk allele have lower trough methadone plasma levels (p<0.05). *ABCB1* carriers of the risk allele did not have an affect on peak methadone plasma levels. *ABCB1* carriers	No statistically significant differences in response to treatment when comparing carriers and non carriers of the (T) risk allele, p>0.2.
						of the risk allele TT genotype had a 0.8 fold (0.7–1) decrease in (R) methadone plasma level, and 0.7 fold (0.6–0.9) decrease in (s) methadone in comparison to non carriers (p = 0.01).	
Fonseca, 2011 [20]	rs1045642	42% (T)	Response to treatment and methadone pharmacokinetics	**Methadone Plasma Concentration Genotype Analysis:** 1­way ANOVA with tukey post hoc analysis **MMT Response Genotype Analysis:** Chi Square	CC: 97 [15–270] CT: 102 [25–400] TT: 91 [15–190]	No significant difference in methadone plasma concentration for *ABCB1* risk allele (T) carriers, p>0.05	No statistically significant association found for carriers of T risk allele among responders and non responders genotyped for the *ABCB1* rs1045642 SNP, p = 0.266.
Hung, 2011 [30]	rs1045642	Low Dose (<55 mg): 40.76% Medium Dose (55–99 mg): 40% High Dose (100–150 mg):	Methadone dose	Comparison of methadone dose in carriers and non– carriers using an odds ratio.	Low Dose: (<55 mg) CC: 32.61 CT: 53.26 TT: 14.13 Medium Dose: (55–99 mg) CC: 36 CT: 48 TT: 16	n/a	n/a
		63.92%			High Dose: (100–150 mg) CC: 11.39 CT: 49.37 TT: 39.24 Carriers of the risk allele (T) show an association with higher methadone dose than non-carriers (p<0.0001).		
Levran, 2008 [21]	rs1045642	Low Dose Methadone Subjects: 36% (T) High Dose Methadone Subjects: 51% (T) Overall, 45% (T)	Methadone dose (genotype frequencies among high and low dose patient groups)	Comparison of high vs low methadone dose using an odds ratio (p = 0.054)	High Dose (N = 53) CC: 30% CT: 43% TT: 26% Low Dose (N = 44) CC: 27% CT: 64% TT: 9%	n/a	n/a
Uehlinger, 2007 [48]	rs1045642	50% (T)	The effect of quetiapine on methadone metabolism. Data for methadone plasma concentration is	Mann–Whitney U Test	n/a	No statistically significant differences in methadone plasma concentrations in carriers and non- carriers of the T risk	n/a
			reported for all subjects prior to and after consumption of quetiapine.			allele (p>0.05).	

**Table 5:** Summary of Findings Table for Outcome Data in *CYP2B6* Studies.

First Author Last Name, Year of Publication	SNPs	Minor Allele Frequency	Outcome(s) Assessed	Statistical Measures of Association	Methadone Dose Among Genotyped Groups	Response to Treatment (% yes)	Results
Crettol, 2005 [18]	*6 (*9 rs3745274	24%%, *6	Methadone	Kruskal Wallis was used for	n/a	5 (2.3–10)	**Methadone Plasma**
	*4 rs2279343)		pharmacokinetics and	the comparison of			**Concentration:**
			response to MMT	concentrations of R, S, RS			p = 0.0004
			treatment	methadone among patients			
				with different genotypes			*CYP2B6*was found to be
				(carriers of *6 versus non			influenced by the *6 allele,
				carriers). This statistical			with carriers having an
				measure was also used		38 (30–47)	increased S methadone
				when comparing responders			plasma concentration
				and non­responders.			p = 0.004.
						56 (48–65)	**Methadone Response:**
							p>0.05
							Carriers of allelic variant
							*6 did not have different
							rates of MMT response
							than non­carriers.
Crettol, 2006 [17]	*6 (*9 rs3745274 *4 rs2279343)	24%, *6	Methadone kinetics (methadone plasma levels) and response to treatment	Log transformed data used for comparison between genotype and phenotype groups with independent t­ test/1 way ANOVA for linear regression	n/a	n/a	**Methadone Plasma Concentration:** p = 0.0001 Homozygous carriers of *6 allele were found to have significantly higher trough (s) methadone plasma levels (p = 0.0001) compared to heterozygous and non­carriers of *6. There was also a trend toward higher trough R levels for carriers of *6 allele (p = 0.07).
Fonseca, 2011 [20]	*6 (*9 rs3745274	24%, *6	Methadone	1­way ANOVA with tukey	Homozygous *6	57	**Methadone Plasma**
	*4 rs2279343)		pharmacokinetics and	post hoc analysis to asses	Carriers:		**Concentration:**
			response to MMT.	both genotypic differences in	74 (24)		p>0.05
				response to treatment and			No association but a
				methadone plasma	Non­Carriers *6:		trend toward higher
				concentrations.	100 (65)	5	(S) methadone plasma
							concentrations among
							homozygous carriers of
							*6 allele
Levran, 2008 [21]	*6 (*9 rs3745274 *4 rs2279343)	Not reported	Methadone dose		n/a		
Uehlinger, 2007 [48]	*6 (*9 rs3745274 *4 rs2279343)	39%, *6	The effect of quetiapine on methadone metabolism	Mann­Whitney U Test	n/a	n/a	Their primary question did not focus on Methadone plasma concentration differences based on genetic profile.

Only observational studies investigating patients on MMT for the purpose of treating opioid addiction were included in the systematic review. The observational studies needed to have: a) investigated one of the SNPs of interest AND b) looked at this SNP in relation to one of the outcomes of interest (i.e. methadone dose, methadone plasma level, or continued illicit opioid use). No age or sex restriction was placed on the study populations.

Eligible studies included in this systematic review must have been performed in human study populations. There were no restriction dates on publications, however for the purpose of time and resources, unpublished literature was not included in this review. Observational studies examining opioid addiction patients using buprenorphine or a substitute opioid therapy (SOT) other than methadone were not eligible for inclusion. However, for studies examining the genetic predisposition for treatment response outcomes in patients on methadone in comparison to patients on a different SOT, only the data on the MMT patients were extracted. In addition, pilot studies or incomplete studies were not eligible for full data abstraction.

The prevalence of genetic association studies in the literature has increased exponentially over the past decade. Most are cross-sectional studies that present unique methodological challenges and risks of bias and therefore such studies were appraised accordingly when included in systematic reviews and meta-analyses. To assess the risk of bias for individual studies in this investigation, a modified Newcastle Ottawa Scale (NOS) instrument was used [39]. We removed several categories highlighting the comparability of cohort or case/control selection and the importance of adequate follow-up between study groups, while also introducing categories that emphasize explicit outcomes and genetic assessment. To review the instrument, please refer to Table S4.

All observational studies meeting the inclusion criteria for full text extraction were subjected to a methodological quality assessment. The two raters (Dennis, B and Bawor, M) independently assessed the methodological quality of each article using an amended Newcastle-Ottawa Scale for observational studies [39]. A third party (Samaan, Z) was called in to resolve any disagreements that arose during the methodological rating process. All studies that met a high risk of bias ranking (13.5/27) for the amended cross-sectional genetic risk of bias tool or a high risk of bias ranking of 4 stars or less for the modified Newcastle Ottawa Scale for case control study research, were subjected to further subgroup analysis when the data were combined to assess whether or not differences in the results can be explained by differences in methodological quality. This is a slight modification from the original protocol, which anticipated only using the Newcastle Ottawa scale for case-control/cohort studies.

To understand whether or not we can be confident in the significant estimates, we constructed a series of GRADE evidence profiles in an effort to rank the quality of evidence presented in the summary statistics.

Due to the variability of how MMT response is defined in the literature, this review has looked at multiple MMT response outcomes. The MMT response outcomes focused on in this review include: methadone dose, continued illicit opioid abuse, and methadone metabolism or methadone plasma concentrations. Currently, methadone dose is defined in the literature as an average or maximum daily dose during the first year of treatment, where it is treated as a continuous variable that can be measured through self-report or chart review in mg/day [17], [18], [21], [22], [30], [33], [40], [41]. Methadone metabolism is defined as plasma concentrations in methadone patients measured during the steady-state in the trough (R), (S), and (RS) forms, where it is a continuous variable measured in ng*kg/mL [8], [17], [18], [20], [24]. Methadone response is most commonly measured as a binary variable (separated by responders and non-responders), as determined by abstinence from opioids during a period generally spanning a time greater than 2 months, where it can be measured through self-report and/or urine drug toxicology screening [8], [17], [39]–[43]. See Table 1 for a detailed description of how these outcomes are currently defined, measured, and statistically analyzed within genetic research.

Full text extraction forms were created for the purpose of this systematic review and are available upon request. Information abstracted from the individual studies includes; study design, number of participants, ethnicity of participants, genes assessed, SNPs assessed, methadone outcome, statistical measurement, statistical association, p-values, confidence intervals, handling of missing data, handling of multiple testing error, and information on covariates tested in each model. Any disagreements that arose during the data extraction stage were resolved by discussion between the raters, or if no solution was reached, by consulting a third party (Samaan, Z). All studies eligible for full text extraction are presented in detail in Table 3.

The results of this study are synthesized in both a narrative and statistical manner. We conducted several meta-analyses using a random-effects model to address each of the outcomes of interest; methadone dose, methadone trough (R) and (S) plasma concentration, and response to methadone treatment defined as abstinence from illicit substance abuse. All statistical analyses were completed using Review Manager 5.0. For the meta analyses pooling the results from studies investigating the association between the *ABCB1* (rs1045642) SNP and the CYP2B6*6 haplotype and trough (R) and (S) methadone plasma concentrations, the standardized mean difference between genotypes was used. The standardized mean difference was also used when pooling the results of studies investigating the association between the SNPs of interest and methadone dose. When pooling results of studies investigating methadone response by genotype, we were able to use a dichotomous outcome and use response as an event among participants, resulting in a pooled odds ratio for the included studies. Assessing for publication bias among the pooled studies using Egger's plot for each of the forest plots generated for this review resulted in limited findings, arguably due to the small sample size and limited number of studies eligible for pooling.

When combining results of the dichotomous data (response to treatment) into a summary odds ratio, we implemented the Mantel-Haenszel method, in which the model is able to estimate between study variation through an evaluation of each study's final results to a Mantel-Haenszel fixed effect meta analysis result. This is a random effects approach available through Review Manager, Version 5 [42].

Each SNP and its associated MMT outcome of interest are displayed in separate forest plots. All studies suitable for inclusion in the meta-analysis are weighted by the inverse of the variance. Presenting the data in forest plots with the associated confidence intervals allows us to also determine whether or not there is heterogeneity in the results, however we will need to address the a priori hypotheses about heterogeneity to determine the possible reasons for result differences across studies. We anticipate possible differences between studies based on influencing factors such as outcome measurement and study design. It is also known that allelic frequencies are influenced by ethnicity. In addition, some variability is anticipated based on the study quality (i.e. design, methodological score from Newcastle Ottawa Scale and outcome measurement). For example, some studies may use self-report to determine concomitant opioid abuse, while other studies may use urine toxicology screening to determine this outcome. Hence, the possible differences in the continued opioid abuse (response) outcome results between studies may be explained by the influence of social desirability bias. An I^2^ statistic has been used to determine whether there is heterogeneity in the results of the studies or if the actual difference in the results is attributable to chance alone [43]. An I^2^ test statistic of 40% or greater is considered to be an indication of the presence of important heterogeneity among studies [42].

## Results

The search was performed from inception of databases to April 1^st^ 2013. After applying theoutlined search strategy defined in Table S3 to the Medline, EMBASE, CINAHL, PsycINFO, and Web of Science databases, we obtained an initial yield of 182 articles after duplicate screening. Please refer to Figure 1 for a detailed flow diagram outlining the article screening process. Please refer to Table 3 for a list of the selected studies. Of the studies that entered the full text screening (n = 12) [17], [18], [20], [21], [24], [30], [33], [44]–[48], only seven were eligible for data abstraction [17], [18], [20], [21], [30], [33], [48]. In 100% of the excluded cases (n = 5) the studies had limited reporting in their abstract about the single nucleotide polymorphisms being reviewed, where in the full text review the screeners discerned that the articles did not look at either of the SNPs of interest.

The observed quadratic weighted kappa agreement between the two independent raters for the title, abstract, and full text screening was found to be 0.82 (95% CI 0.7, 0.95), 0.43 (95% CI 0.01, 0.85), and 0.43 (95% CI 0.01, 0.85), respectively. The decline in agreement for the abstract and full-text screening is arguably due to the limited number of studies available for screening in the later phases.

### i) Study Characteristics

The study designs found in this review were cross-sectional (n = 5) and case-control (n = 2). The studies included in this review were performed on a predominantly male patient population, with the percentage of males ranging from 65.3 to 78.8 percent (Table 3). Among the seven included studies, all but one study [30] were performed in a majority Caucasian population. The mean age of participants across studies was comparable with a range from 32.1 to 45 years of age (Table 3).

A total of seven studies were reviewed to determine the association between the *ABCB1* (rs1045642) and *CYP2B6* (*9 rs3745274, *4 rs2279343) genetic polymorphisms and patient response to methadone maintenance therapy (Table 4 and Table 5). Among the seven articles, three investigated the association between the *ABCB1* genetic polymorphism (rs1045642) and trough (R) and (S) methadone plasma concentrations. While we were able to extract data on five articles investigating the association between the CYP2B6*6 haplotype (*9 rs3745274, *4 rs2279343) and trough (R) and (S) methadone plasma concentrations, one of the articles was an interim analysis [18], thus we were unable to include this study when we later pooled the results in a meta-analysis. Among the articles reviewing the association between the CYP2B6*6 haplotype or the *ABCB1* (rs1045642) SNP and continued illicit substance abuse among MMT patients, only two articles provided data on each gene of interest [17], [20]. When reviewing articles addressing the relationship between genotypes and methadone dose, three articles [17], [18], [20] were located for the CYP2B6*6 haplotype and four articles [17], [21], [30], [33] for the *ABCB1* (rs1045642) SNP which provided data.

### ii) Risk of Bias Assessment

When evaluating the risk of bias in cross-sectional genetic studies (n = 5) [17], [18], [20], [21], [48], a number of studies were limited by reporting quality (Table S5). Five (100%) of the cross sectional studies evaluated did not report whether or not there was blinding during the outcome or exposure assessment (kappa = 1.0). In addition to the poor reporting quality surrounding the blinding of assessors when discerning the outcome or exposure, there was also poor reporting quality for the genetic methodological analysis section, with only 40% (n = 2) of studies explicitly detailing high quality methods for genetic analysis (i.e. good call rate, Hardy-Weinberg equilibrium criteria fulfillment). It is important to also note that of these studies (n = 2) with good reporting and quality in genetic analysis methodology, one is an interim analysis of the other, meaning that the second study is the completed genetic analysis of the first with a greater number of participants and different planned genotyping analysis.

In addition, only 60% of the cross-sectional genetic studies had <10% of data missing, meaning only 60% of the cross-sectional studies were ranked with a low risk of bias when evaluated on missing data (kappa = 1.0). All but one study (Fonseca, 2011) had a detailed description of their outcome measurements, such as trough (R) and (S) plasma concentration assessment using plasma analysis or urine toxicology screening to asses illicit substance abuse/response to treatment (kappa = 1.0). All but one study (Uehlinger, 2007), had study samples that were representative of the cohort of interest (n = 4), where the Uhelinger (2007) study was limited by strict inclusion/exclusion criteria due to the strict outcome being assessed (effect of quetiapine on methadone dose). When assessing whether the cohorts were drawn from the same population, 60% of studies were able to fulfill the criteria for low risk of bias, as demonstrated by patients being selected from a similar population (i.e. time, and similar place of residence). The studies that failed to meet this criteria (Crettol 2005, 2006), were limited by their larger dispersion of source MMT sites due to their inability to adjust for other confounding variables which could be influenced by site of administration (i.e. socioeconomic status, physician variability in prescribing practice).

When assessing whether studies controlled for important confounding variables such as concurrent medication, concurrent treatment, body mass index (BMI), or duration of MMT, 60% (n = 4) of the cross-sectional studies evaluated had a low risk of bias [17], [18], [20], [48].

The modified Newcastle Ottawa scale was also used to asses the two case control studies that were evaluated during this review [30], [33] (Table S6). Of the two studies evaluated only one (Hung, 2011) was considered to be at low risk of bias, receiving a score of 8/9 stars. One limitation of the Hung (2011) study was their inability to adequately report whether cases and controls had the same method of assessment for the genetic risk allele (exposure), due to the lack of explicit reporting of the genotyping methods for both cases and controls. The second case control study (Coller, 2006) was evaluated as being subject to a high risk of bias, receiving a low score of 4/9 stars. The Coller (2006) study had very limited reporting quality for the sections considered in the assessment, including; definitions of cases, selection of participants, and statistical adjustment.

### iii) The Impact of the *ABCB1* (rs1045642) Genetic Polymorphism on Methadone Dose, Metabolism, and Patient Response

#### A) Trough (R) Methadone Plasma Concentration

Our first meta-analysis (Figure S1 and S2) pooled results of studies examining the association between the *ABCB1* (rs1045642) genetic polymorphism and the trough (R) methadone plasma concentrations. The SMD is the difference in mean effects across the comparator genotype groups divided by the pooled standard deviation of participants mean trough (R) methadone plasma concentrations. In this meta-analysis we see a comparison of the mean trough (R) methadone plasma concentration between participants with the *ABCB1* (rs1045642) CC versus TT genotypes and CC versus CT genotypes. To estimate the effect of the standardized mean difference many refer to Cohen's (1988) statistical criteria, which dictates that a standardized mean difference of 0.2 represents a small effect, 0.5 a moderate effect, and 0.8 a large effect [49]. In the meta-analyses of trough (R) methadone plasma concentrations (Figure S1 and S2), while not significant, we see a small effect of 0.23, p = 0.41 (95% CI, −0.31, 0.77, Figure S1) and 0.07, p = 0.61 (95% CI, −0.19, 0.33, Figure S2), for the pooled results of CC versus TT and CC versus CT respectively, indicating that trough (R) methadone plasma concentrations do not differ widely amongst participants with different genotypes. There was minimal observed heterogeneity among the pooled results for the comparisons of the CC versus TT and CC versus CT respectively (I^2^ = 41%, I^2^ = 0%).

#### B) Trough (S) Methadone Plasma Concentration

In the second grouping of meta-analyses we see a comparison of the mean trough (S) methadone plasma concentration between participants with the *ABCB1* (rs1045642) CC versus TT genotypes (Figure S3) and CC versus CT genotypes (Figure S4). In the meta-analyses of trough (S) methadone plasma concentrations, while not significant, we again see small effects of 0.3, p = 0.22 (95% CI, −0.19, 0.81, Figure S3) and 0.17, p = 0.41 (95% CI, −0.23, 0.56, Figure S4), for the pooled results of CC versus TT and CC versus CT respectively, indicating again that trough (S) methadone plasma concentration does not differ based on this SNP genotype. There was minimal observed heterogeneity among these pooled results for the comparisons of the CC versus TT and CC versus CT respectively (I^2^ = 36%, I^2^ = 35%).

#### C) Response to Methadone Maintenance Therapy

In the third set of meta-analyses, we are comparing the differences in response to treatment (illicit drug use) among participants with the *ABCB1* CC versus TT genotypes (Figure S5) and CC versus CT genotypes (Figure S6). Within this analysis the number of events are considered the number of participants who have “responded” to methadone maintenance therapy, meaning the number of participants who are abstaining from illicit substances. A resulting non-significant odds ratio of 0.86, p = 0.81 (95% CI, 0.25, 2.95, Figure S5) and 0.85, p = 0.7 (95% CI, 0.37, 1.95, Figure S6) is observed for the comparison of the CC versus TT and CC versus CT genotypes respectively. These results indicate that there is no difference in methadone response between participants with different genotype profiles for the rs1045642 SNP. The confidence in the estimates for this meta-analysis is low, largely due to the heterogeneity among pooled studies, where both I^2^ calculations exceeded 51%.

#### D) Methadone Dose

Our final grouping of meta-analyses for the *ABCB1* SNP pooled results of studies examining the association between the *ABCB1* (rs1045642) polymorphism and methadone dose. In the meta analyses of methadone dose, while not significant, we see a large effect of 0.76, p = 0.24, (95% CI, −0.50, 2.01, Figure S7) and −0.02, p = 0.88, (95% CI,- 0.27, 0.23, Figure S8), for the pooled results of CC versus TT (Figure S7) and CC versus CT (Figure S8) respectively, indicating that methadone dose is not significantly associated with this genetic variant. There was a considerable amount of heterogeneity observed among the pooled results for the comparisons of the CC versus TT (I^2^ = 92%), but not the CC versus CT genotypes (I^2^ = 0%).

### iv) The Impact of the CYP2B6*6 Haplotype (*9 rs3745274, *4 rs2279343) on Methadone Dose, Metabolism and Patient Response

#### A) Trough (R) Methadone Plasma Concentration

**Figure 2:**
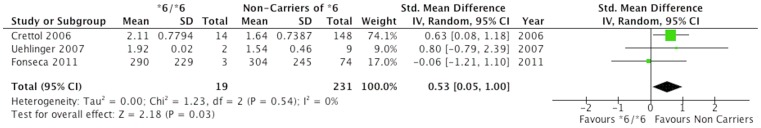
CYP2B6 (Homozygous *6 Carriers Versus Non-Carriers of *6) Trough (R) Methadone Plasma Concentrations.

**Figure 3:**
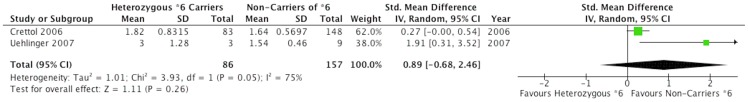
CYP2B6 (Heterozygous *6 Carriers Versus Non-Carriers of *6) Trough (R) Methadone Plasma Concentrations.

We compared the mean trough (R) methadone plasma concentration between homozygous *6 and non *6 carriers as well as the heterozygous *6 and non *6 carriers of the *CYP2B6*(*9 rs3745274, *4 rs2279343) *6 haplotype. In the meta-analyses determining the association between the *6 haplotype and trough (R) methadone plasma concentration, we see a significant SMD between genotyped groups. When comparing homozygous carriers of the *6 haplotype with non-carriers (Figure 2), we see an SMD effect of 0.53, p = 0.03, (95% CI, 0.05,1.00) trending toward a large difference. Participants genotyped as homozygous *6 carriers are seen to have trough (R) methadone plasma concentrations higher than non-carriers of the *6 haplotype, suggesting that homozygous *6 carriers metabolize methadone at a slower rate than non-carriers of the haplotype. Comparing heterozygous carriers of the *6 haplotype with non-carriers (Figure 3), we see an SMD effect of 0.89 p = 0.26, (95% CI, −0.68, 2.46), which can be considered by Cohen's criteria as a large effect, however not statistically significant but a directionally consistent finding in this analysis. We again see a considerable range of observed heterogeneity among these pooled results for the comparisons of the *6 homozygous and *6 heterozygous carriers versus non-carriers of the *6 haplotype with I^2^ values of 0 and 75 percent respectively.

#### B) Trough (S) Methadone Plasma Concentration

**Figure 4:**
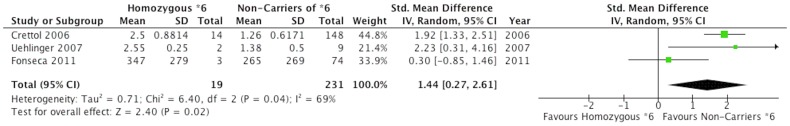
CYP2B6 (Homozygous *6 Carriers Versus Non *6 Carriers) Trough (S) Methadone Plasma Concentrations.

**Figure 5:**
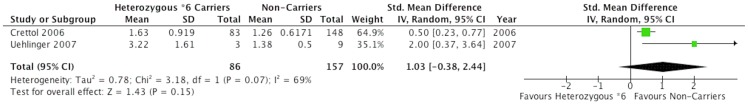
CYP2B6 (Heterozygous *6 Carriers Versus Non-Carriers of *6) Trough (S) Methadone Plasma Concentrations.

In the meta-analyses determining the association between the *6 haplotype and trough (S) methadone plasma concentration, we see a significant SMD between genotyped groups. When comparing homozygous carriers of the *6 haplotype with non-carriers (Figure 4), we see an SMD effect of 1.44, p = 0.02, (95% CI, 0.27,2.61), a considerably large effect difference. Participants genotyped as homozygous *6 carriers are seen to have trough (S) methadone plasma concentrations higher than non-carriers of the *6 haplotype, suggesting that homozygous *6 carriers metabolize methadone at a significantly slower rate than non-carriers of the haplotype. However, in the meta analysis comparing heterozygous carriers of the *6 haplotype with non-carriers (Figure 5), we see an SMD effect of 1.03, p = 0.15 (95% CI,-0.38, 2.44), which can be considered by Cohen's criteria as a large effect, however not significant in this analysis, but directionally consistent with homozygous *6 carriers versus non-carriers. Subsequently, we again see a large amount of observed heterogeneity among these pooled results for the comparisons of the *6 homozygous and *6 heterozygous carriers versus non-carriers of the *6 haplotype with I^2^ values of 69 percent for both analyses.

#### C) Response to Methadone Maintenance Therapy

When comparing the differences in response to treatment (illicit drug use) among participants who are homozygous carriers of the *6 haplotype against participants who are non-carriers of this haplotype (*1/*1), we see a resulting non-significant odds ratio of 1.05, p = 0.93 (95% CI, 0.37 2.97), indicating that response to treatment is not affected by the presence or absence of the *6 haplotype (Figure S9). Within this analysis the number of events are considered the number of participants who have “responded” to methadone maintenance therapy, meaning the number of participants who are not continuing to abuse illicit substances. Observed heterogeneity in this meta analysis was minimal, with an I^2^ value of zero percent.

#### D) Methadone Dose

In this final meta-analysis, we see a comparison of the mean methadone dose between homozygous *6 carriers and non *6 carriers of the CYP2B6*6 (*9 rs3745274, *4 rs2279343) haplotype (Figure S10). When comparing homozygous carriers of the *6 haplotype to non-carriers, we see an SMD effect of −0.21, p = 0.37 (95%CI,-0.68, 0.26), which can be considered by Cohen's criteria as a very small effect. This analysis while not significant, does show a trend that indicates that carriers of the *6 variant are more likely to have lower doses then participants without the *6 haplotype. As shown earlier, carriers of the *6 haplotype have been demonstrated to have higher (R) and statistically significant higher (S) methadone plasma concentrations, indicating that the individuals with the *6 haplotype are slower metabolizers of methadone. The analysis above indicates there is a trend for *6 carriers to have lower methadone doses, which is consistent with the findings that these *6 carriers are slower metabolizers, hence their requirement for lower doses of methadone for a stable drug effectiveness. Observed heterogeneity in this meta-analysis was minimal, with an I^2^ value of 0 percent.

## Discussion

Genetic variants are known to influence an individuals' vulnerability to develop a disorder such as addiction [50]; in addition to also influencing a patient's response to therapy [30]. Research aimed at determining the influence of genetic factors on drug metabolism is termed pharmacogenetic research and has been evolving for the last 20 years [51]. A large focus of pharmacogenetic research is currently centered in areas such as cardiovascular disease and oncology, however, the study of genetic impact on individual response to MMT remains underdeveloped. When attempting to understand why the current state of genetic research on methadone response is limited, one may begin to consider reasons such as the limitations in participant population. The recruitment, retention in treatment, and follow-up potential of methadone patients has serious limitations, largely due to the transient and impulsive nature of an opioid addiction patient population [52]. In addition, many of the available studies are fraught with methodological limitations including; small sample size, poor genetic call-rates indicating weak genotyping methods, limited reporting quality, and high risk of missing data due to the large loss to follow-up with this patient population. It is important to note that there is currently limited literature available to conduct a meta-analysis to determine the effect of genetic polymorphisms on methadone response.

After an extensive investigation of the genetic determinants of individual patient response to MMT, we only found seven studies that investigated a SNP of interest within a patient population on methadone treatment for opioid addition in relation to one of the outcomes of interest (i.e. methadone dose, methadone plasma level, or continued illicit opioid abuse).

Individually, these studies suggest that 1) carriers of the *ABCB1* (rs1045642) risk allele (T) do not significantly differ from patients without this risk allele on outcomes (Table 4). The meta analyses support the individual study findings, with no significant differences by genotype for the *ABCB1* (rs1045642) SNP for all outcomes of interest. However, one study by Crettol et al. (2006) suggests that carriers of the T allele metabolize methadone at a faster rate than non-carriers (p<0.01) [17]. A primary objective of this systematic review was to determine whether the contention in the literature was due to real observed differences in the outcomes of interest or differences in methodological quality. In an effort to better understand the meta-analyses presented on the *ABCB1* genotype, we looked into the study presented by Crettol (2006). This study was the largest contributor to magnitude of association witnessed in each meta-analysis (including the *CYP2B6* series of analyses), requiring us to look further into the risk of bias this study posed and inevitably influence our confidence in the estimates.

The Crettol (2006) study was not limited by a serious risk of bias during the methodological quality review where it received a ranking of 20/27 (Table S5). The major limitations affecting the Crettol (2006) study was their inability to account for blinding of assessors during the exposure and outcome assessment periods. However, it is not anticipated that a lack of blinding would influence the risk of bias in this study, primarily due to the objective nature of the outcome measurement for both methadone plasma concentration and dose. The other main limitations are the small sample size and risk for type 1 error. Another important study requiring attention is by Uehlinger et al. (2007), arguably due to the inconsistency witnessed between the Uehlinger (2007) results and the other results of the pooled studies. A large limitation of the Uehlinger (2007) study was the small sample size (n = 14), with some estimates comparing genotype groups with only 3 participants in each (i.e. TT, n = 3 versus CC, n = 3). In addition, the primary objective in the Uehlinger (2007) investigation was not to determine the influence of genotype on methadone response, but actually the influence of genotype in quetiapine metabolism within methadone patients.

When determining the influence of *ABCB1* genetic variants on methadone dose, the literature has consistently presented us with negative findings, which we witness again in this systematic review. However, recent research suggests that the large inter-individual variability in methadone dose may be influenced by an interactions between *ABCB1* polymorphisms and p-glycoproteins [53]. Most recent investigations into this area are limited by the lack of available clinical populations; further evidence is required to examine this interaction.

Individually these studies also suggest that 1) The CYP2B6*6 haplotype carriers metabolize methadone at a significantly slower rate than non-carriers and 2) the CYP2B6*6 haplotype does not influence methadone dose or response to treatment (Table 5). The meta-analyses reviewing the influence of genetic polymorphism of *CYP2B6* on methadone response are consistent with the individual studies. While carriers of the *6 haplotype were not found to have different illicit substance abuse behaviors or doses than non-carriers, they were found to metabolize methadone at a significantly slower rate than non-carriers (p = 0.02). However, it is important to be cautious of the significant estimates presented in the *CYP2B6* series of meta-analyses, due to the poor confidence we have in the obtained estimates.

Results from this systematic review suggest that the *CYP2B6* haplotype may influence methadone plasma concentration, however it is not found to be associated with drug doses or clinical outcomes. These results may be due to the influence of other *CYP450* genetic variations involved in the methadone N demethylation processes. For example, the *CYP3A4/5*, and *CYP2D6* genetic polymorphisms are known variants that may also contribute to this reaction [20]. Patients expressing the *CYP3A5* variant have been shown to have high levels of *CYP3A5* activity [20]. The *CYP3A5* variant is also known to represent 50% of the total *CYP3A* hepatic content [20]. Thus, interaction among the *CYP450* polymorphisms may be a large influence on the results suggesting a null effect of *CYP2B6* on modifying methadone dose, begging the need for further research into this area.

Invitro studies have consistently demonstrated that the *CYP2B6* genetic variants preferentially metabolize the (S) enantiomer, with S-methadone resulting as a potent inhibitor of R-methadone N- demethylation [54]–[57]. Invivo studies, as well as the present meta-analysis, have indicated the preferential metabolism of the (S) enantiomer [17], [24], [58]. There are several clinical implications for the preferential 11 influence of S-enantiomer metabolism, one being the higher blockage potency of the S-enantiomer toward 12 the hERG channel, a gene coding for the alpha subunit protein of the potassium ion channel involved in 13 mediating electrical cardiac activity [19]. Several Studies have suggested an association between the S 14 enantiomer and prolonged QT interval [19], [58], [59]. A study by Eap et. al (2007) showed that (S) methadone 15 inhibited the hERG (the human *Ethere àe goe go*T Related Gene) current 3.5fold more potently than (R), with homozygous *6 carriers having an increased risk of prolonged QTc interval (odds ratio = 4.5, 95% confidence interval = 1.21–7.7; P = 0.03) [19].

In an effort to understand whether or not we can be confident in the significant estimates in this meta-analysis, we constructed a series of 14 GRADE evidence profiles (Figures S11, S12, S13, S14, S15, S16, S17, S18, S19, S20, S21, S22, S23, S24) [60], in all of which the evidence was ranked as very low. In the majority of the GRADE tables created for this review, the studies were ranked to have a serious risk of bias and imprecision. The reasons that our confidence in the estimates is ranked as very low is due to the nature of the studies included for data extraction; all studies were observational, which on its own merit has an inherent risk of bias due to unequal distribution of known and unknown confounding variables. However, since we were able to establish temporality with an exposure that is genetic, we do not believe the generalcriticisms for the high risk of bias affecting cross-sectional studies remains as important among genetic studies. However, none of the studies reported whether or not the outcome/exposure assessors were blinded to the outcome/exposure status of the participant and not all of the pooled studies adjusted for concurrent medication use, duration on MMT, or BMI.

The tests for heterogeneity were for the most part insignificant. However, there were many cases were one study would have an opposite direction of the magnitude of association in comparison to other studies. However, this was most often the case with the Uehlinger (2007) study, which was discussed earlier as being limited by the small sample size, and in addition this study bears a small weight on the total estimate of effect. When establishing the confidence in the estimates as very low for the outcome of methadone response, we noted that not all of the pooled studies adjusted for concurrent medication use, duration on MMT, or BMI. It is important to consider that dose or metabolism may vary among patients with different BMI's [61], hence the importance of BMI when considering the influence of genetics on methadone dose or metabolism. In addition, outcome measurements for response to treatment differed among studies, some studies used self report while other studies used urine toxicology screening. There was also heterogeneity between studies when classifying the definition of response, where some studies defined response when patients abstained from all illicit substances, not just opioids. Some differences in the odds ratio (OR) direction of effect were observed, particularly the direction of associations observed in the Fonseca (2011) study, which showed an OR favoring the CC genotype for all meta-analyses of methadone response.

While our meta-analyses did bring us to some significant results, it is clear there are major limitations within the studies that ultimately influence our confidence in these estimates. In addition, the association we have found to be significant, that is the association between CYP2B6*6 homozygous carriers and slower methadone metabolism (p<0.05) should be interpreted cautiously, particularly because we did not adjust for multiple testing error using the Bonferroni correction.

Understanding the combination of factors that contribute to inter-individual variability in methadone response is a paramount task when attempting to enhance the treatment and MMT outcomes in opioid addiction patients. Numerous factors influence the inter-individual variability in methadone response including methadone dose (increased response with doses ≥60 mg/day [62]), adherence to treatment, concurrent physical comorbidities, and area of residence [63]. Some studies have suggested that an increase in treatment retention is association with methadone dose of >80 mg/day, age >30 years, social support network in the residence, and no concurrent alcohol use [64]. It is known that an increased methadone dose may put a patient at risk for overdose [9], and/or mortality [65]–[67], however it has been reported to also be associated with stronger treatment retention and better patient response [68].

A major limitation of this systematic review is the selection of studies contributing to magnitude of association, especially since a majority of these studies did not account for the aforementioned factors contributing to patient response (Table S5, S6). Consequently, this large inter-individual variability poses uncertainty among physicians when deciding on an adequate dose of methadone for new patients. The importance of dose is inherent, since a dose too low can allow for breakthrough withdrawal symptoms, which can ultimately influence a patients propensity to relapse [68]. A dose too high can inhibit a person's activities of daily living; one study looked into the strong side effects of methadone, reporting fatigue, headache, and depression [69]. As such, this concept of personalized medicine becomes highly relevant for health care practitioners attempting to optimize dosing strategies and favorable outcomes for MMT patients. Understanding the genotype profiles influencing methadone can allow health care practitioners to adequately adjust dosing, and ultimately help patients reach an appropriate stabilization dose as early as possible.

When reviewing the available literature on both the *ABCB1* (rs1045642) and CYP2B6*6 haplotype, it is important to address the possibility that these genes may have complimentary roles in the metabolic disposition of methadone, where one is involved in metabolite formation and another in metabolite elimination or drug absorption. Due to the limited available information, and lack of studies investigating both genes in tandem, we were unable to explore this is association further. It is plausible an interaction between these genes may exist and should be explored further in a primary investigation.

Investigations on methadone patient health outcomes have consistently demonstrated the importance of understanding high-risk factors for MMT patients. By identifying important risk factors for methadone response, clinicians will be able to properly manage patients more successfully and in some cases prevent mortality. Methadone response is a pertinent issue for the study of opioid addiction and understanding patient characteristics that predict response can: 1) enhance patient centered treatment, 2) prevent risk of death and opioid overdose, 3) reduce health expenditure, 4) impact patient surveillance, 5) promote multi-therapy approaches to addiction, and 6) impact dosing requirements. This systematic review has shown that that *ABCB1* (rs1045642) SNP has no significant effect on methadone metabolism, dose, and response to treatment, while the CYP2B6*6 haplotype has a significant effect on methadone metabolism but a minimal effect on patient response and dose of methadone. While there is inherent importance in this study, mainly because it is the first meta-analysis to be performed on the genetic determinants of methadone response, we still strongly caution the reader that our confidence in these estimates is very low, arguably because of the serious risk of bias and imprecision within all of the meta-analyses.

## Supporting Information

Checklist S1 **PRISMA Checklist.**
(PDF)

Figure S1 **ABCB1 (rs1045642) CC versus TT Trough (R) Methadone Plasma Concentrations.**
(TIFF)

Figure S2 **ABCB1 (rs1045642) CC versus CT Trough (R) Methadone Plasma Concentrations.**
(TIFF)

Figure S3 **ABCB1 (rs1045642) CC versus TT Trough (S) Methadone Plasma Concentrations.**
(TIFF)

Figure S4 **ABCB1 (rs1045642) CC versus CT Trough (S) Methadone Plasma Concentrations.**
(TIFF)

Figure S5 **Influence of ABCB1 Genotype (CC versus TT) on Methadone Maintenance Therapy Response to Treatment.**
(TIFF)

Figure S6 **Influence of ABCB1 Genotype (CC versus CT) on Methadone Maintenance Therapy Response to Treatment.**
(TIFF)

Figure S7 **Influence of ABCB1 Genotype (CC versus TT) on Methadone Dose.**
(TIFF)

Figure S8 **Influence of ABCB1 Genotype (CC versus CT) on Methadone Dose.**
(TIFF)

Figure S9 Influence of CYP2B6 *6 Haplotype on Methadone Maintenance Therapy Response to Treatment.
(TIFF)

Figure S10 Influence of Homozygous *6 Carriers Versus Homozygous *1/*1 Non-carriers on Methadone Dose.
(TIFF)

Figure S11 **GRADE ABCB1 Trough (R) Methadone Plasma Concentration (CC vs TT).**
(TIFF)

Figure S12 **GRADE ABCB1 Trough (R) Methadone Plasma Concentration (CC vs CT).**
(TIFF)

Figure S13 **GRADE ABCB1 Trough (S) Methadone Plasma Concentration (CC vs TT).**
(TIFF)

Figure S14 **GRADE ABCB1 Trough (S) Methadone Plasma Concentration (CC vs CT).**
(TIFF)

Figure S15 GRADE CYP2B6 Trough (R) Methadone Plasma Concentration (Homozygous *6 Carriers Versus Non-Carriers).
(TIFF)

Figure S16 GRADE CYP2B6 Trough (R) Methadone Plasma Concentration (Heterozygous *6 Carriers Versus Non-Carriers).
(TIFF)

Figure S17 GRADE CYP2B6 Trough (S) Methadone Plasma Concentration (Homozygous *6 Carriers Versus Non-Carriers).
(TIFF)

Figure S18 GRADE CYP2B6 Trough (S) Methadone Plasma Concentration (Heterozygous *6 Carriers Versus Non-Carriers).
(TIFF)

Figure S19 **GRADE ABCB1 Response to Methadone Maintenance Therapy (Illicit Substance Abuse Behaviors) CC vs TT.**
(TIFF)

Figure S20 **GRADE ABCB1 Response to Methadone Maintenance Therapy (Illicit Substance Abuse Behaviors) CC vs CT.**
(TIFF)

Figure S21 GRADE CYP2B6 Response to Methadone Maintenance Therapy (Illicit Substance Abuse Behaviors) Homozygous *6 Carriers Versus Non-Carriers.
(TIFF)

Figure S22 **GRADE ABCB1 Methadone Dose Requirements by Genotype (CC vs TT).**
(TIFF)

Figure S23 **GRADE ABCB1 Methadone Dose Requirements by Genotype (CC vs CT).**
(TIFF)

Figure S24 GRADE CYP2B6 Methadone Dose Requirements by *6 Genotype.
(TIFF)

Table S1 **The Genetic Determinants for MMT Response.**
(DOCX)

Table S2 **Outline of PICO Questions for Individual SNPs and Outcomes.**
(DOCX)

Table S3 **Full Search Strategy for Systematic Review and Meta-Analysis on the Genetic Determinants of Methadone Maintenance Therapy Response.**
(DOCX)

Table S4 **Modified Newcastle Ottawa Scale Tool to Asses Risk of Bias in Cross-Sectional Genetic Research.**
(DOCX)

Table S5 **Risk of Bias Table for Individual Cross-Sectional Genetic Studies.**
(DOCX)

Table S6 **Risk of Bias Table using the Modified Newcastle Ottawa Scale for Genetic Case Control Studies**.
(DOCX)
