# Supplementary figures and images for: Impact of ABCB1 and CYP2B6 Genetic Polymorphisms on Methadone Metabolism, Dose and Treatment Response in Patients with Opioid Addiction: A Systematic Review and Meta-Analysis
Source: PLoS One. 2014 Jan 29;9(1):e86114. doi: 10.1371/journal.pone.0086114 (PMC3906028; doi:10.1371/journal.pone.0086114)

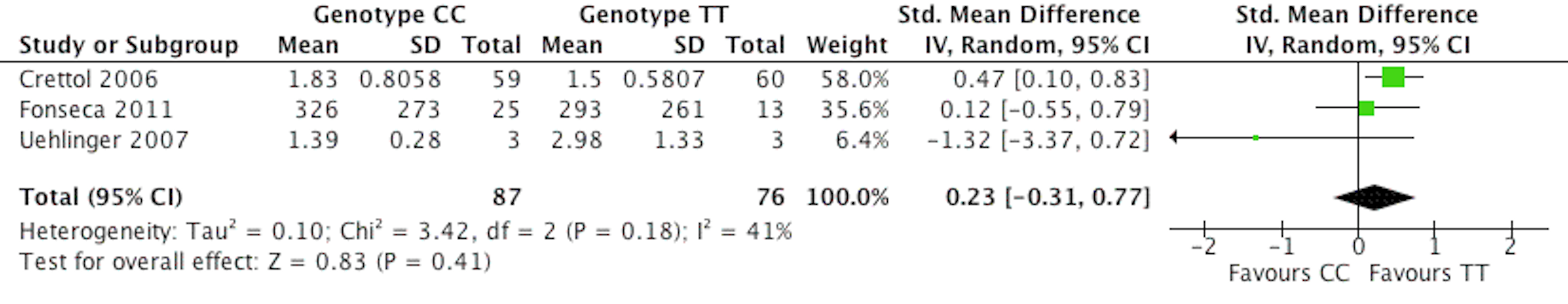

Supplement: Figure S1 — ABCB1 (rs1045642) CC versus TT Trough (R) Methadone Plasma Concentrations. (TIFF) [file pone.0086114.s002.tiff]

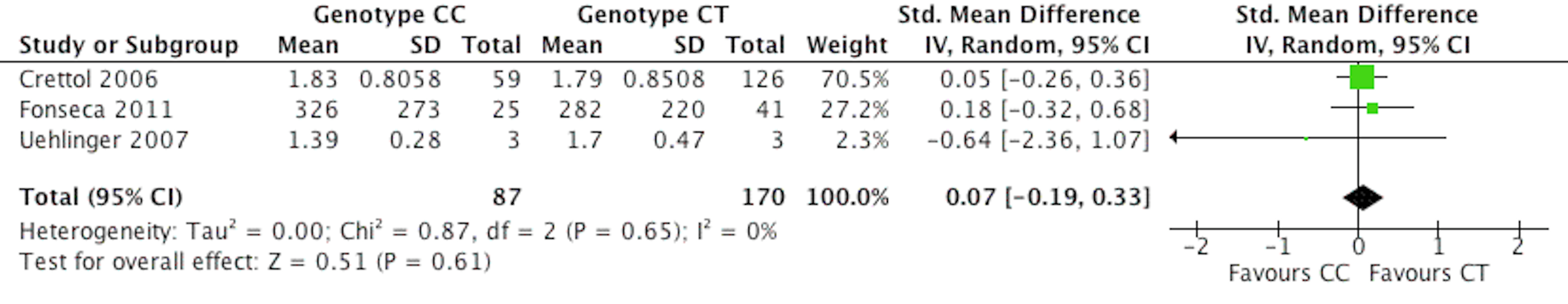

Supplement: Figure S2 — ABCB1 (rs1045642) CC versus CT Trough (R) Methadone Plasma Concentrations. (TIFF) [file pone.0086114.s003.tiff]

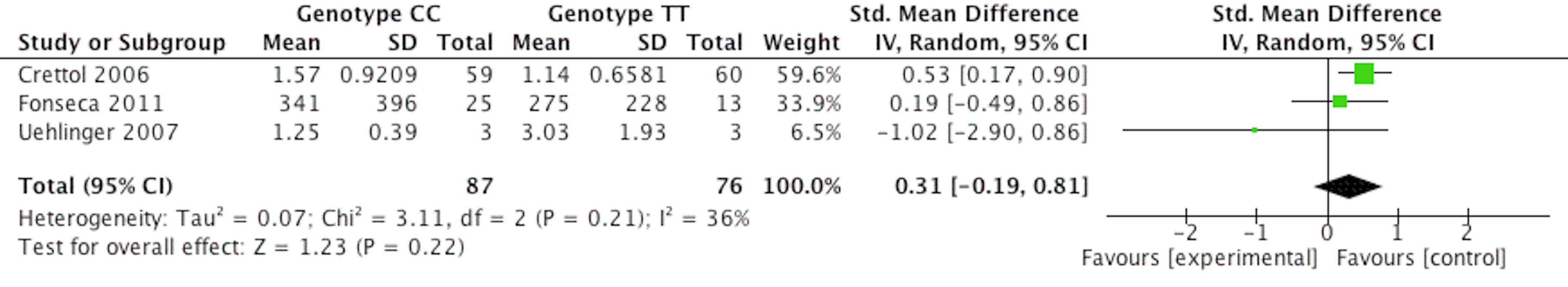

Supplement: Figure S3 — ABCB1 (rs1045642) CC versus TT Trough (S) Methadone Plasma Concentrations. (TIFF) [file pone.0086114.s004.tiff]

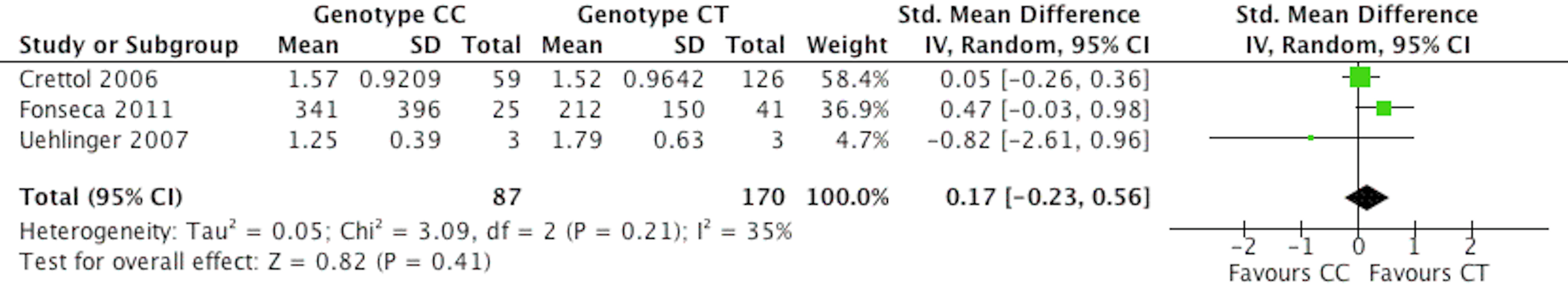

Supplement: Figure S4 — ABCB1 (rs1045642) CC versus CT Trough (S) Methadone Plasma Concentrations. (TIFF) [file pone.0086114.s005.tiff]

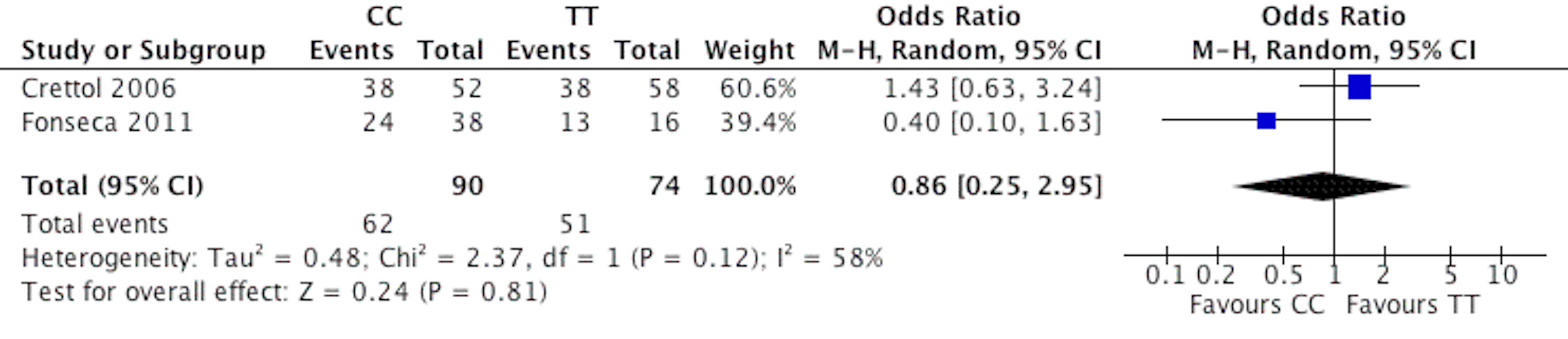

Supplement: Figure S5 — Influence of ABCB1 Genotype (CC versus TT) on Methadone Maintenance Therapy Response to Treatment. (TIFF) [file pone.0086114.s006.tiff]

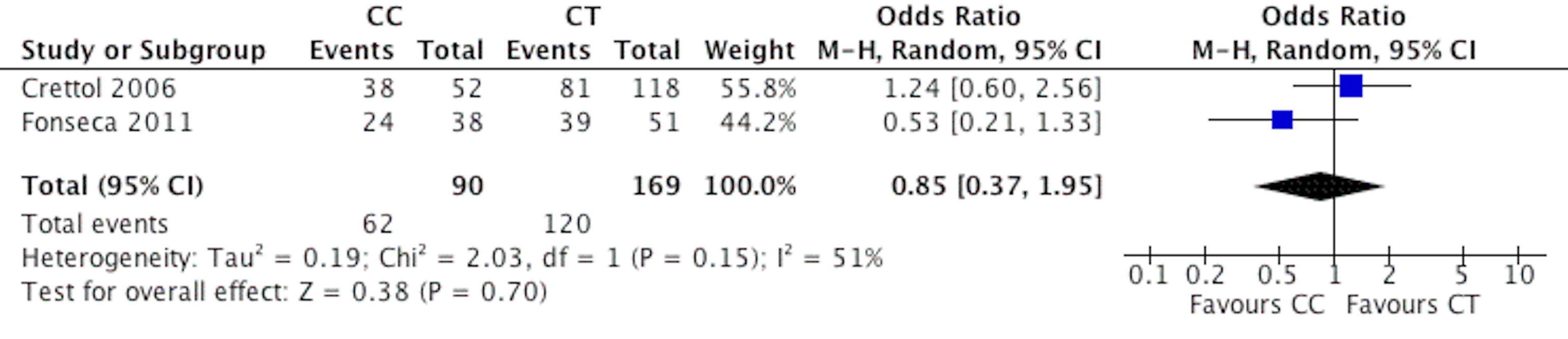

Supplement: Figure S6 — Influence of ABCB1 Genotype (CC versus CT) on Methadone Maintenance Therapy Response to Treatment. (TIFF) [file pone.0086114.s007.tiff]

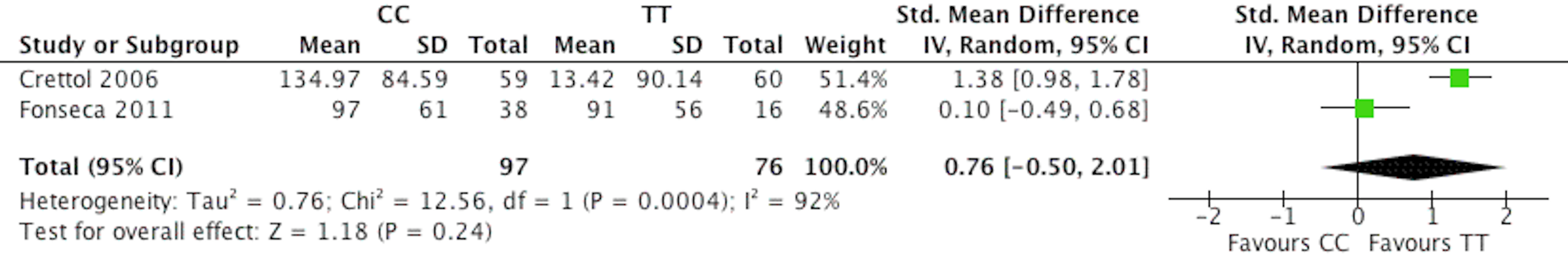

Supplement: Figure S7 — Influence of ABCB1 Genotype (CC versus TT) on Methadone Dose. (TIFF) [file pone.0086114.s008.tiff]

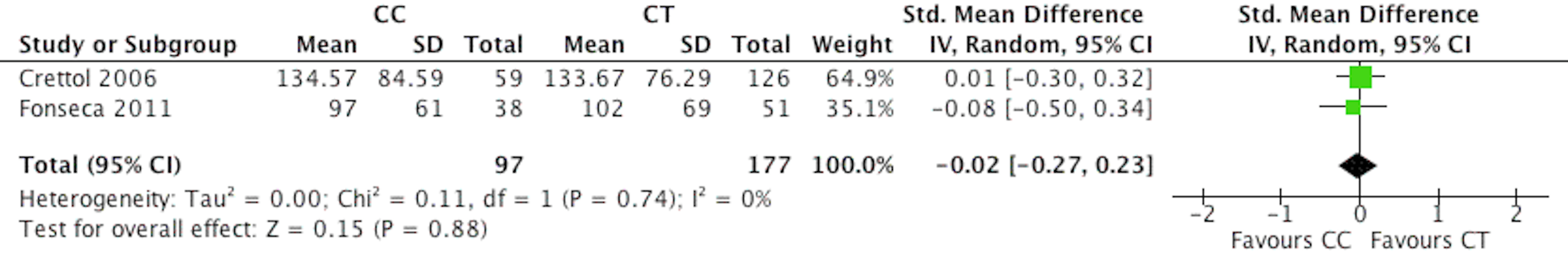

Supplement: Figure S8 — Influence of ABCB1 Genotype (CC versus CT) on Methadone Dose. (TIFF) [file pone.0086114.s009.tif]

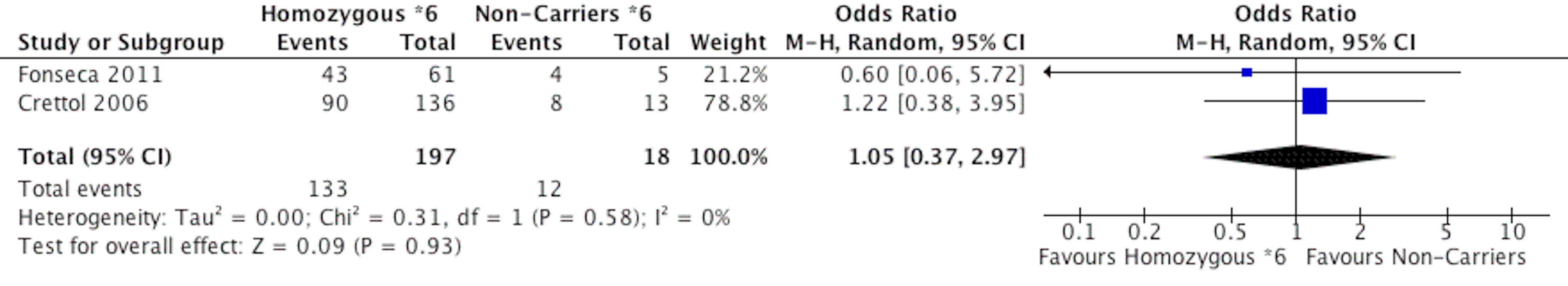

Supplement: Figure S9 — Influence of CYP2B6 *6 Haplotype on Methadone Maintenance Therapy Response to Treatment. (TIFF) [file pone.0086114.s010.tiff]

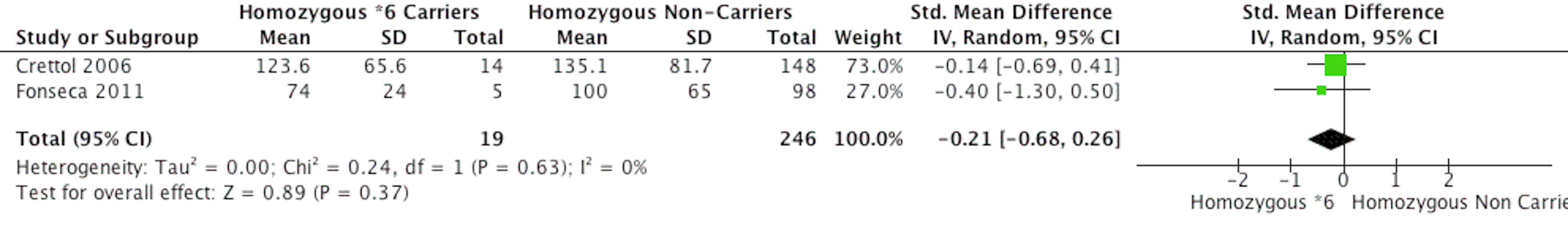

Supplement: Figure S10 — Influence of Homozygous *6 Carriers Versus Homozygous *1/*1 Non-carriers on Methadone Dose. (TIFF) [file pone.0086114.s011.tiff]

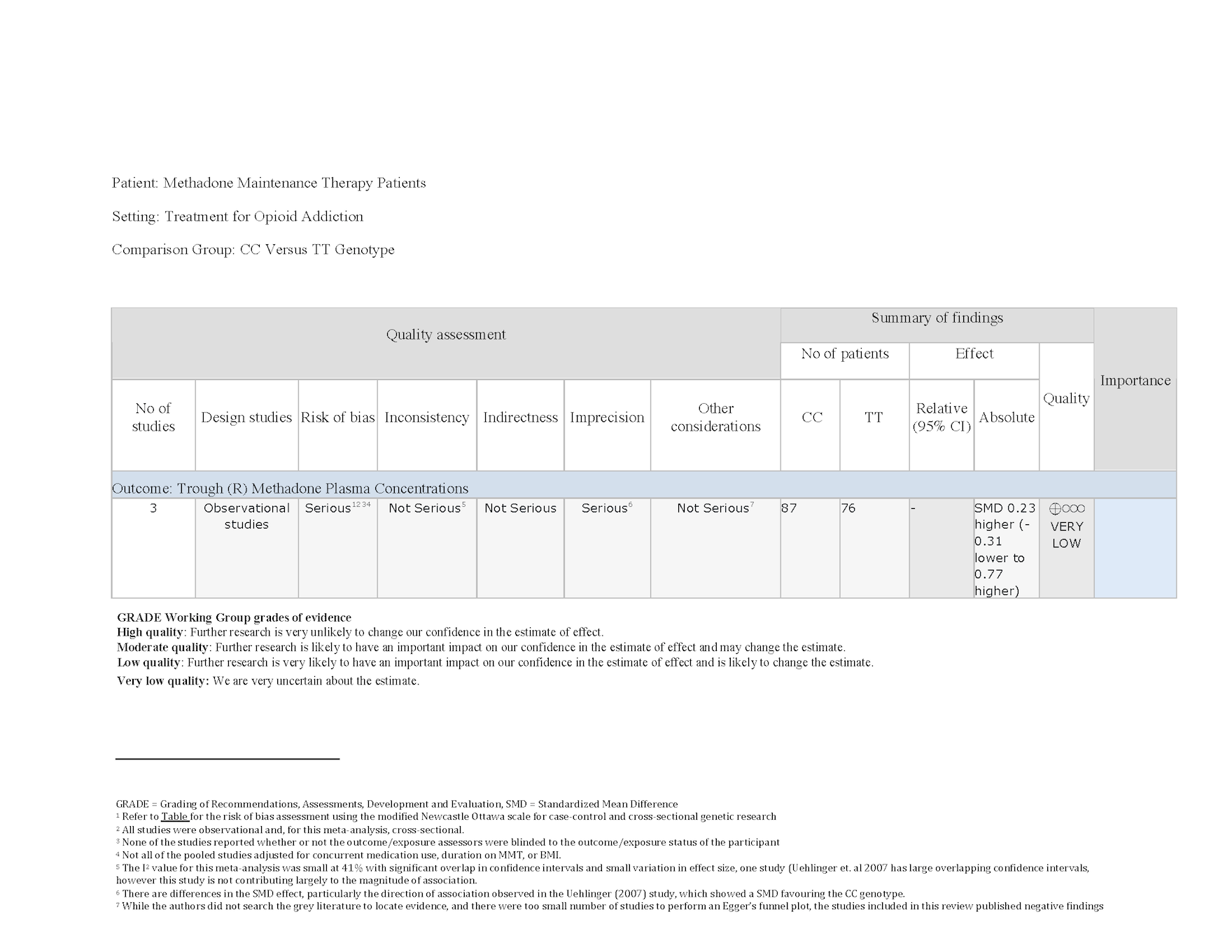

Supplement: Figure S11 — GRADE ABCB1 Trough (R) Methadone Plasma Concentration (CC vs TT). (TIFF) [file pone.0086114.s012.tiff]

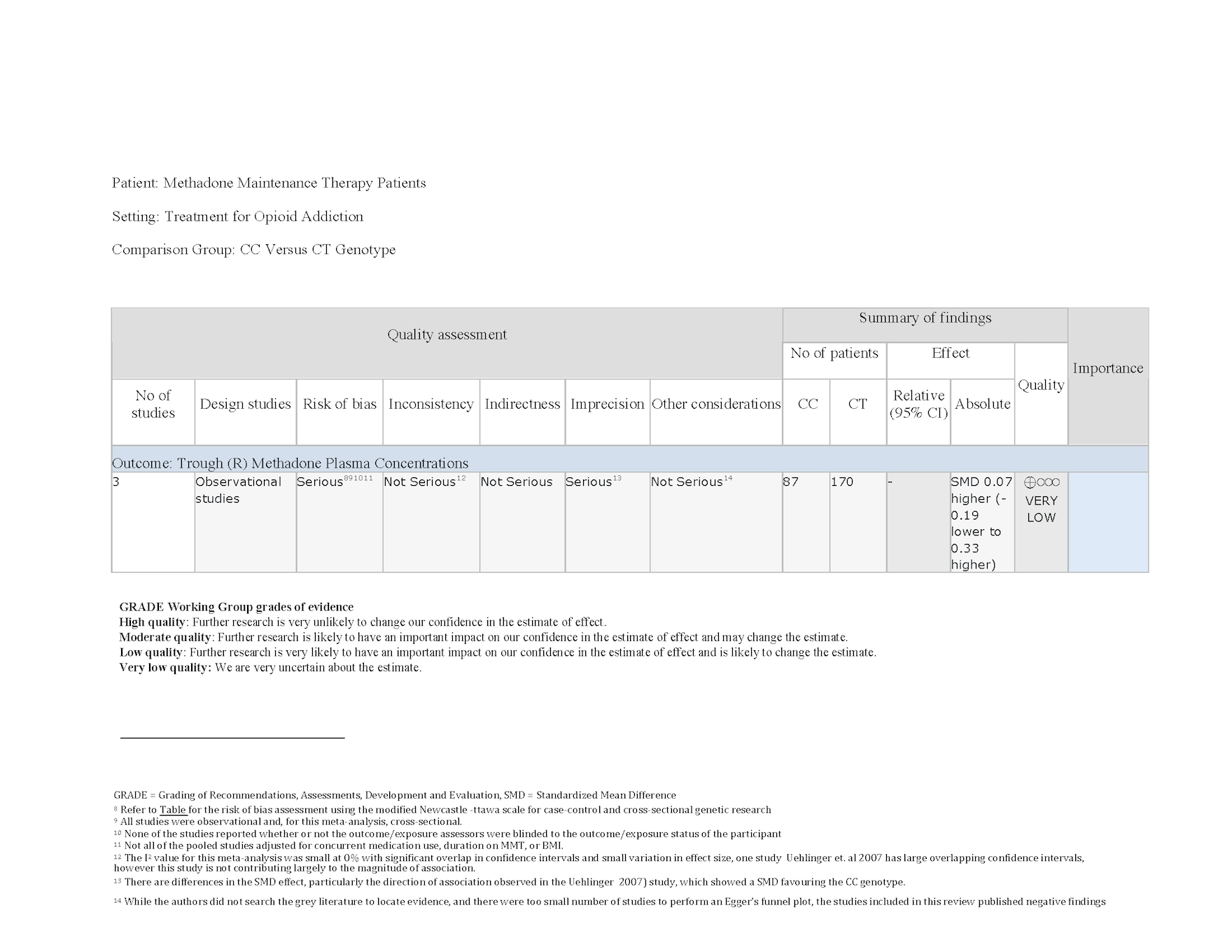

Supplement: Figure S12 — GRADE ABCB1 Trough (R) Methadone Plasma Concentration (CC vs CT). (TIFF) [file pone.0086114.s013.tiff]

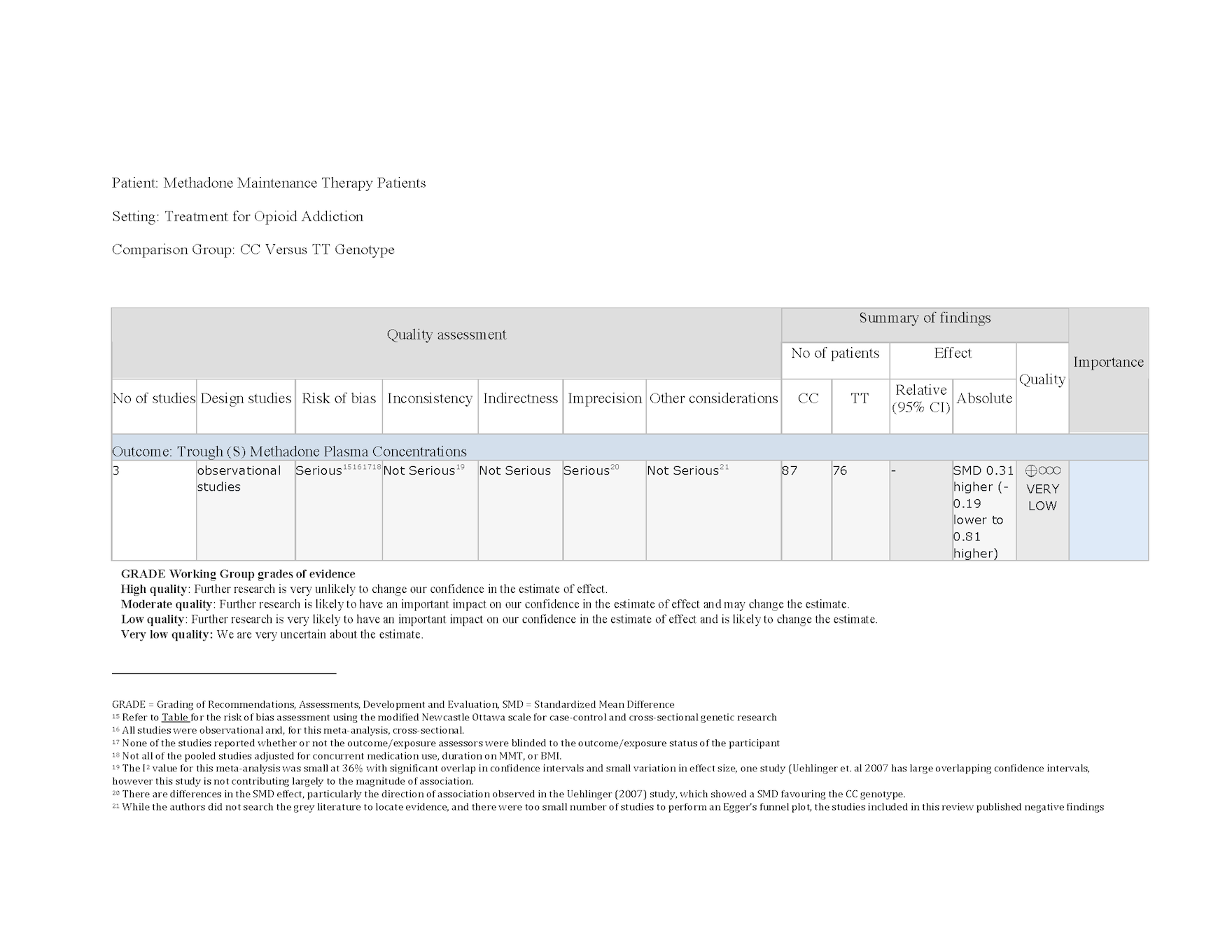

Supplement: Figure S13 — GRADE ABCB1 Trough (S) Methadone Plasma Concentration (CC vs TT). (TIFF) [file pone.0086114.s014.tiff]

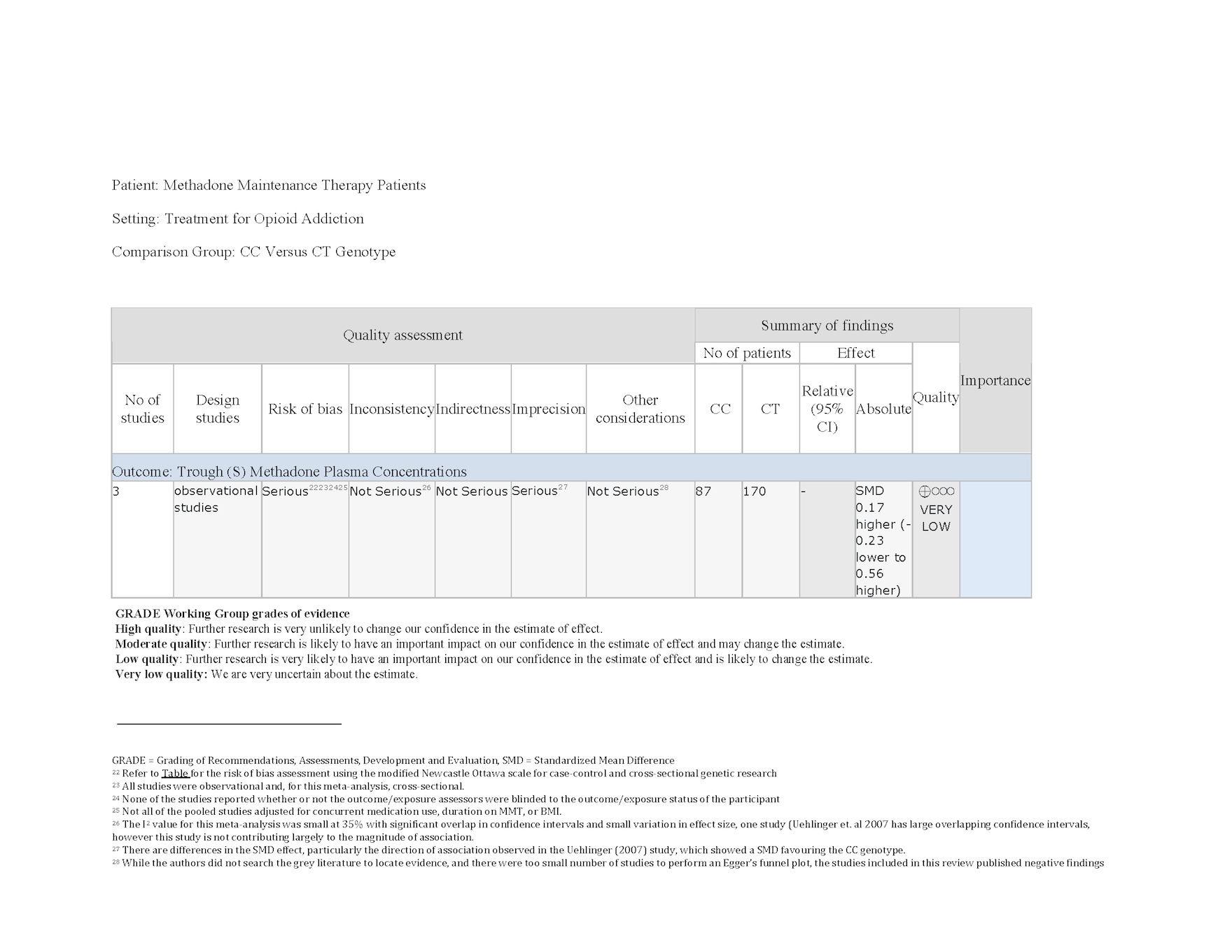

Supplement: Figure S14 — GRADE ABCB1 Trough (S) Methadone Plasma Concentration (CC vs CT). (TIFF) [file pone.0086114.s015.tiff]

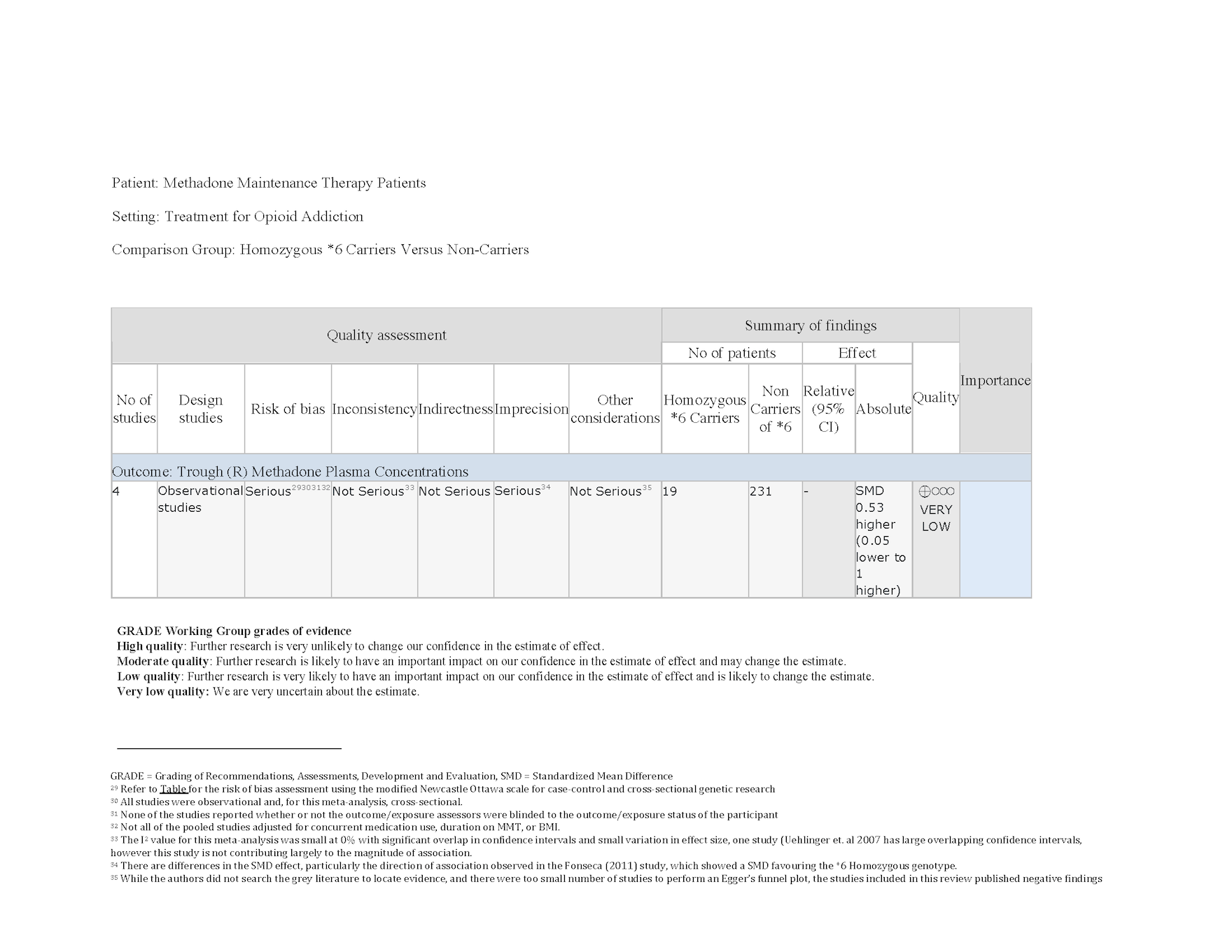

Supplement: Figure S15 — GRADE CYP2B6 Trough (R) Methadone Plasma Concentration (Homozygous *6 Carriers Versus Non-Carriers). (TIFF) [file pone.0086114.s016.tiff]

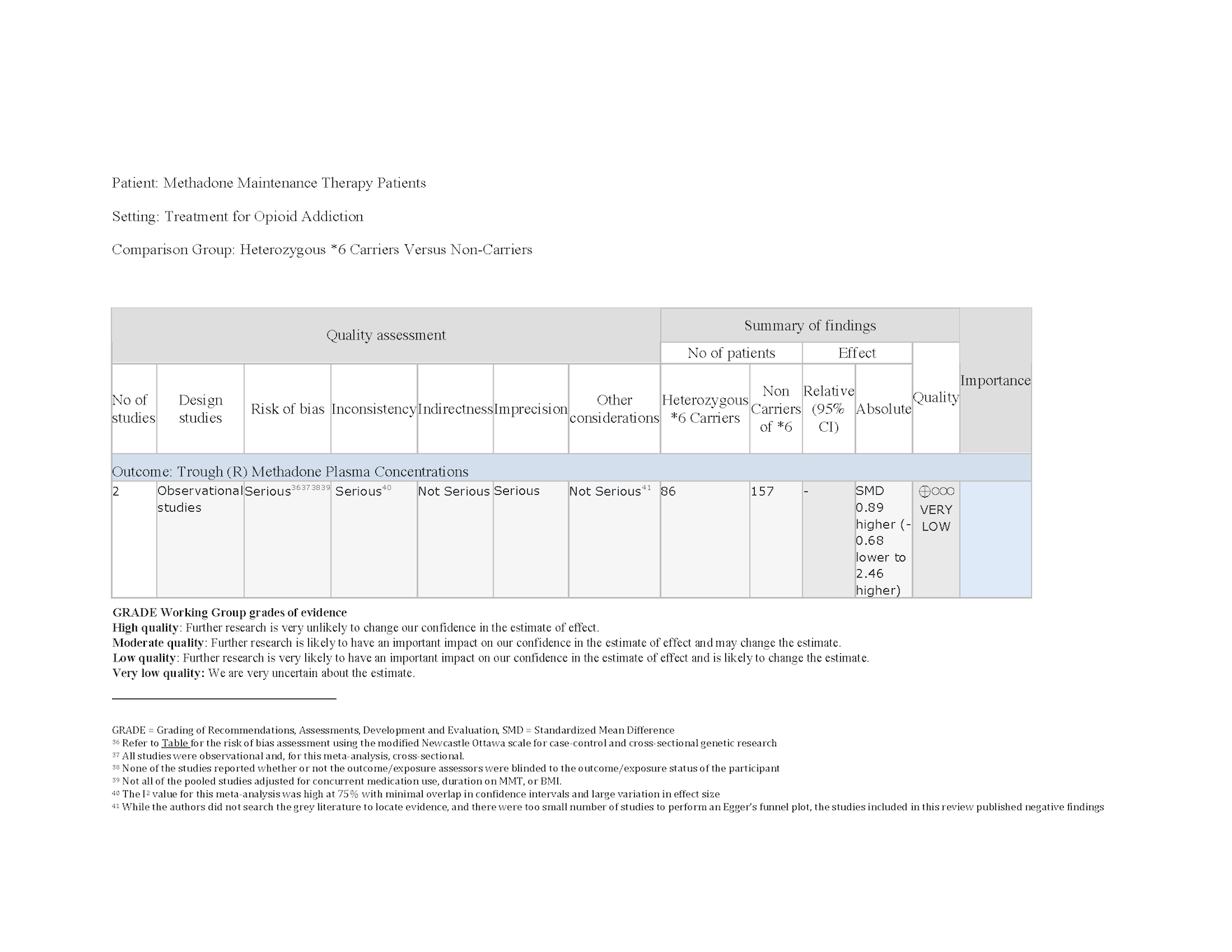

Supplement: Figure S16 — GRADE CYP2B6 Trough (R) Methadone Plasma Concentration (Heterozygous *6 Carriers Versus Non-Carriers). (TIFF) [file pone.0086114.s017.tiff]

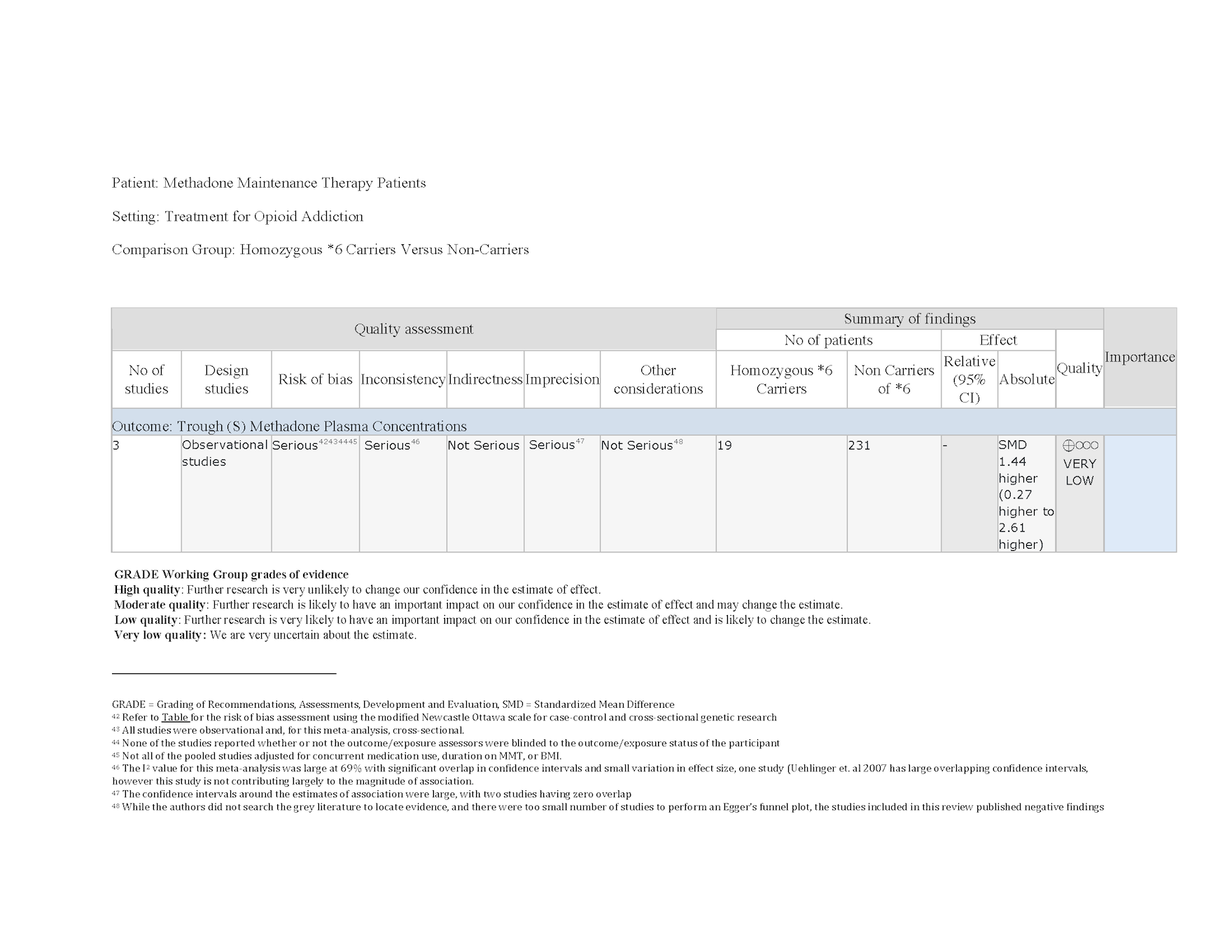

Supplement: Figure S17 — GRADE CYP2B6 Trough (S) Methadone Plasma Concentration (Homozygous *6 Carriers Versus Non-Carriers). (TIFF) [file pone.0086114.s018.tiff]

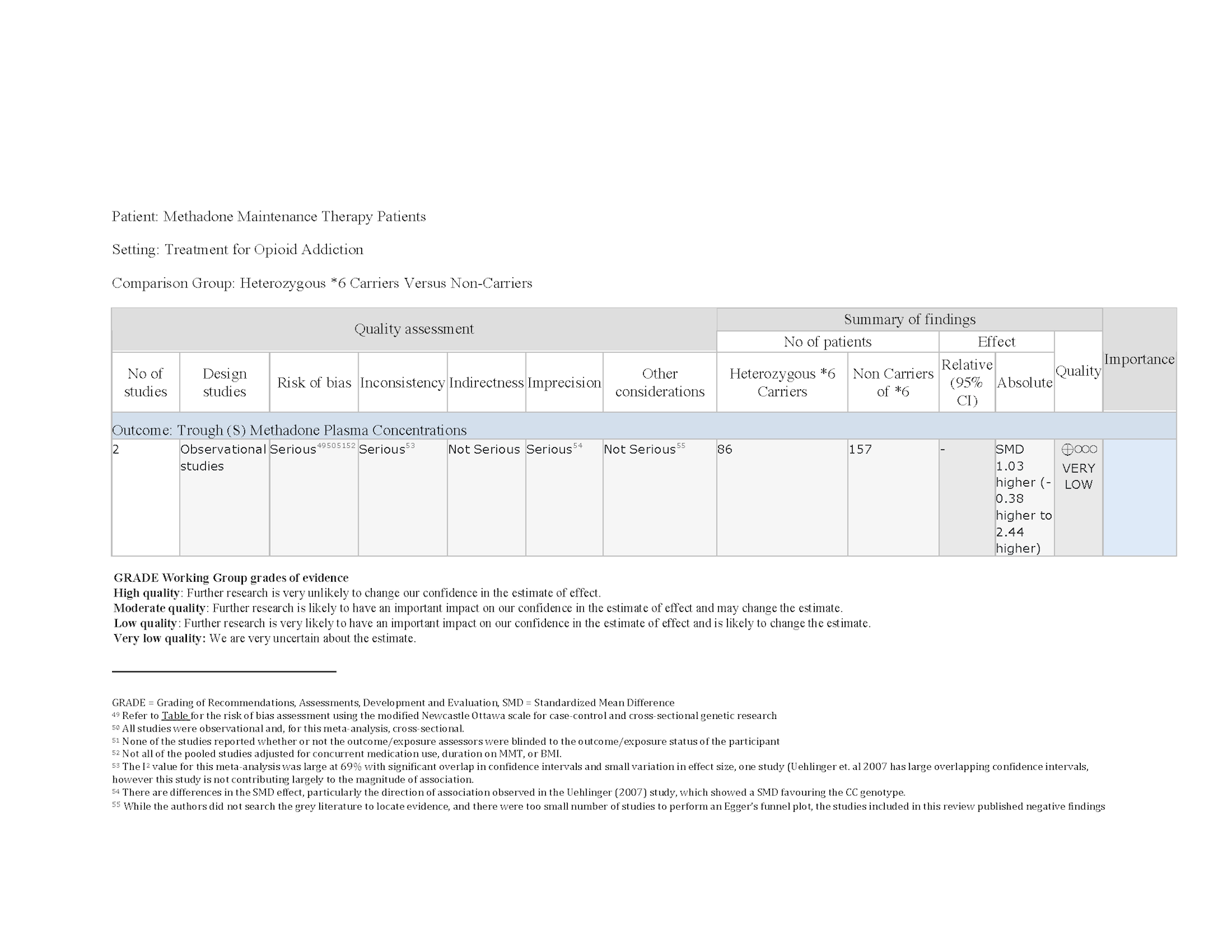

Supplement: Figure S18 — GRADE CYP2B6 Trough (S) Methadone Plasma Concentration (Heterozygous *6 Carriers Versus Non-Carriers). (TIFF) [file pone.0086114.s019.tiff]

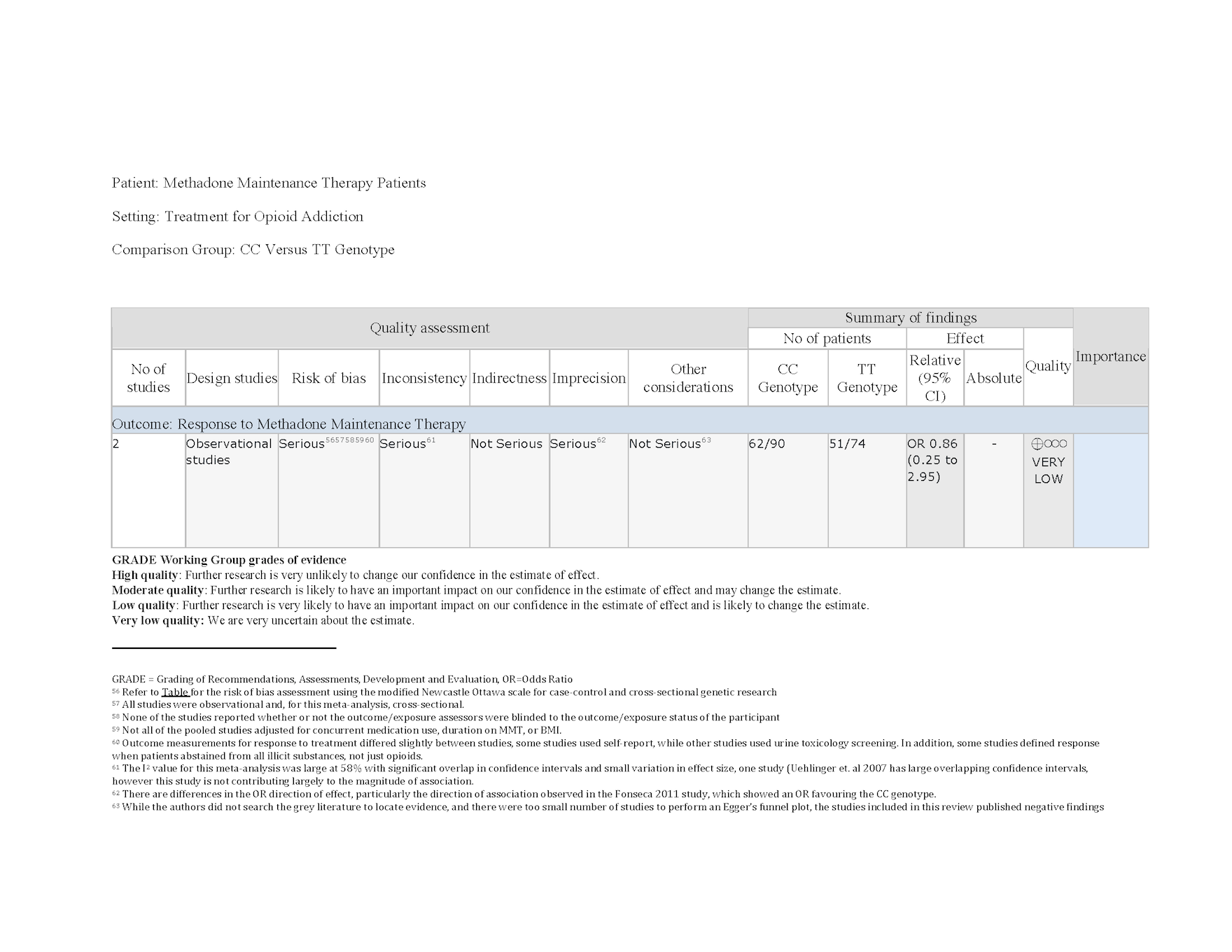

Supplement: Figure S19 — GRADE ABCB1 Response to Methadone Maintenance Therapy (Illicit Substance Abuse Behaviors) CC vs TT. (TIFF) [file pone.0086114.s020.tiff]

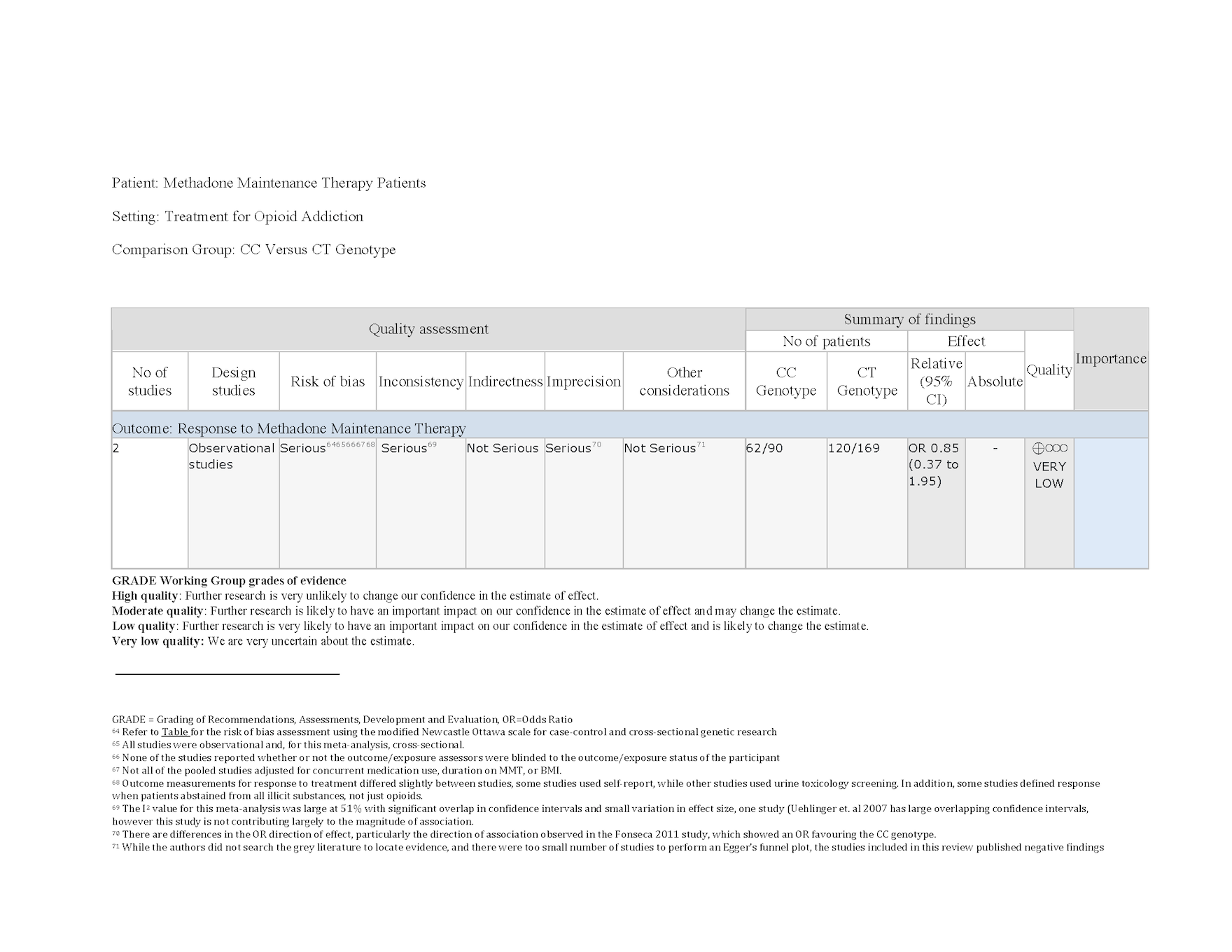

Supplement: Figure S20 — GRADE ABCB1 Response to Methadone Maintenance Therapy (Illicit Substance Abuse Behaviors) CC vs CT. (TIFF) [file pone.0086114.s021.tiff]

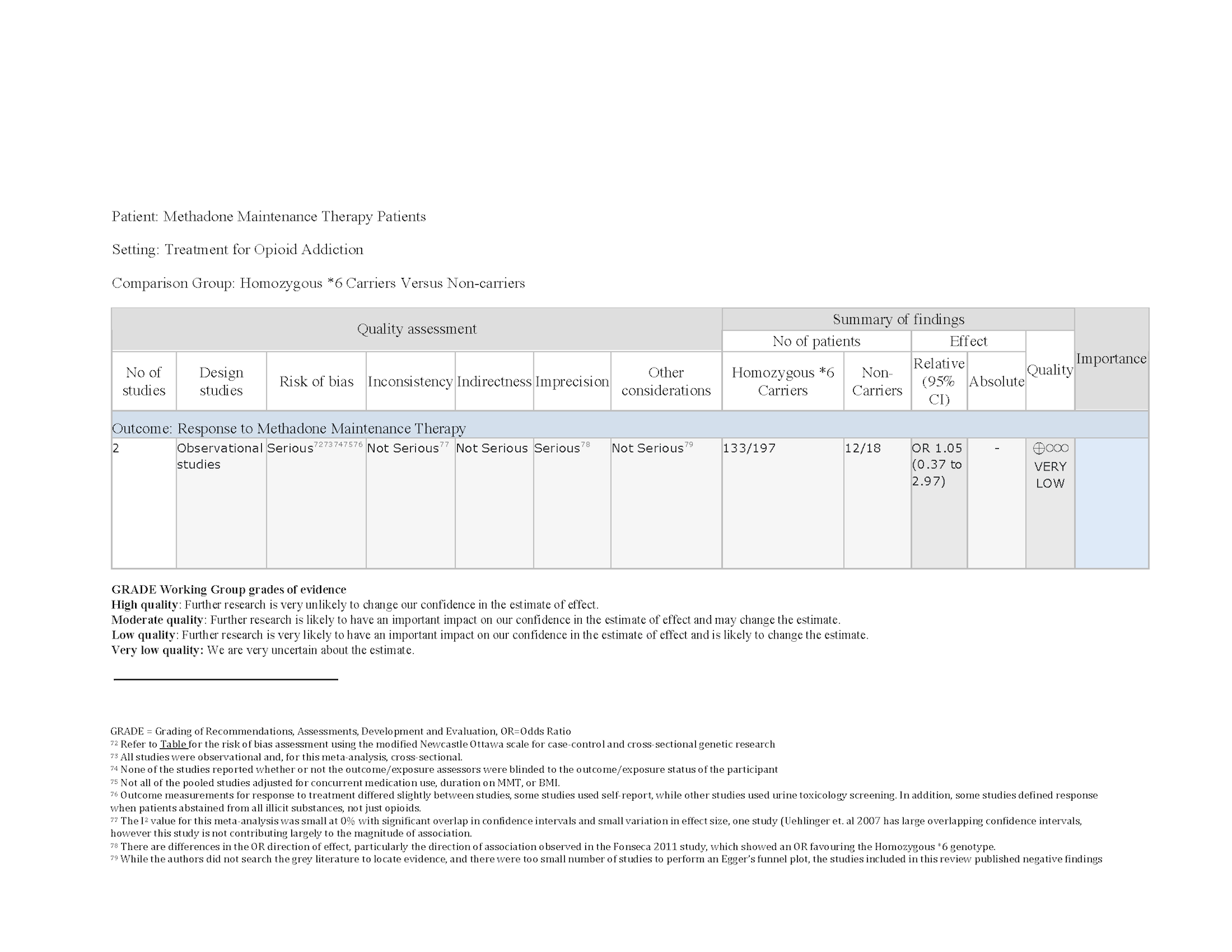

Supplement: Figure S21 — GRADE CYP2B6 Response to Methadone Maintenance Therapy (Illicit Substance Abuse Behaviors) Homozygous *6 Carriers Versus Non-Carriers. (TIFF) [file pone.0086114.s022.tiff]

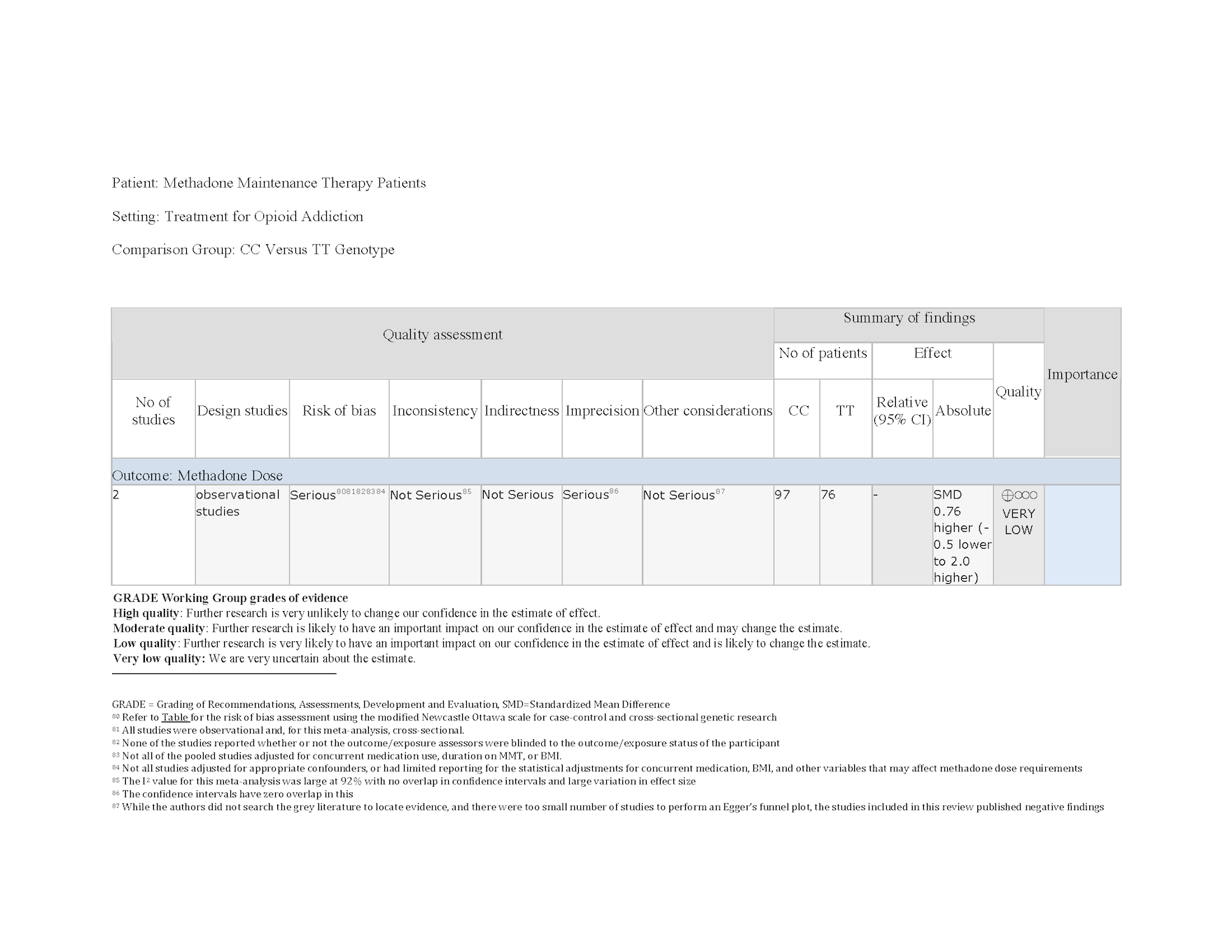

Supplement: Figure S22 — GRADE ABCB1 Methadone Dose Requirements by Genotype (CC vs TT). (TIFF) [file pone.0086114.s023.tiff]

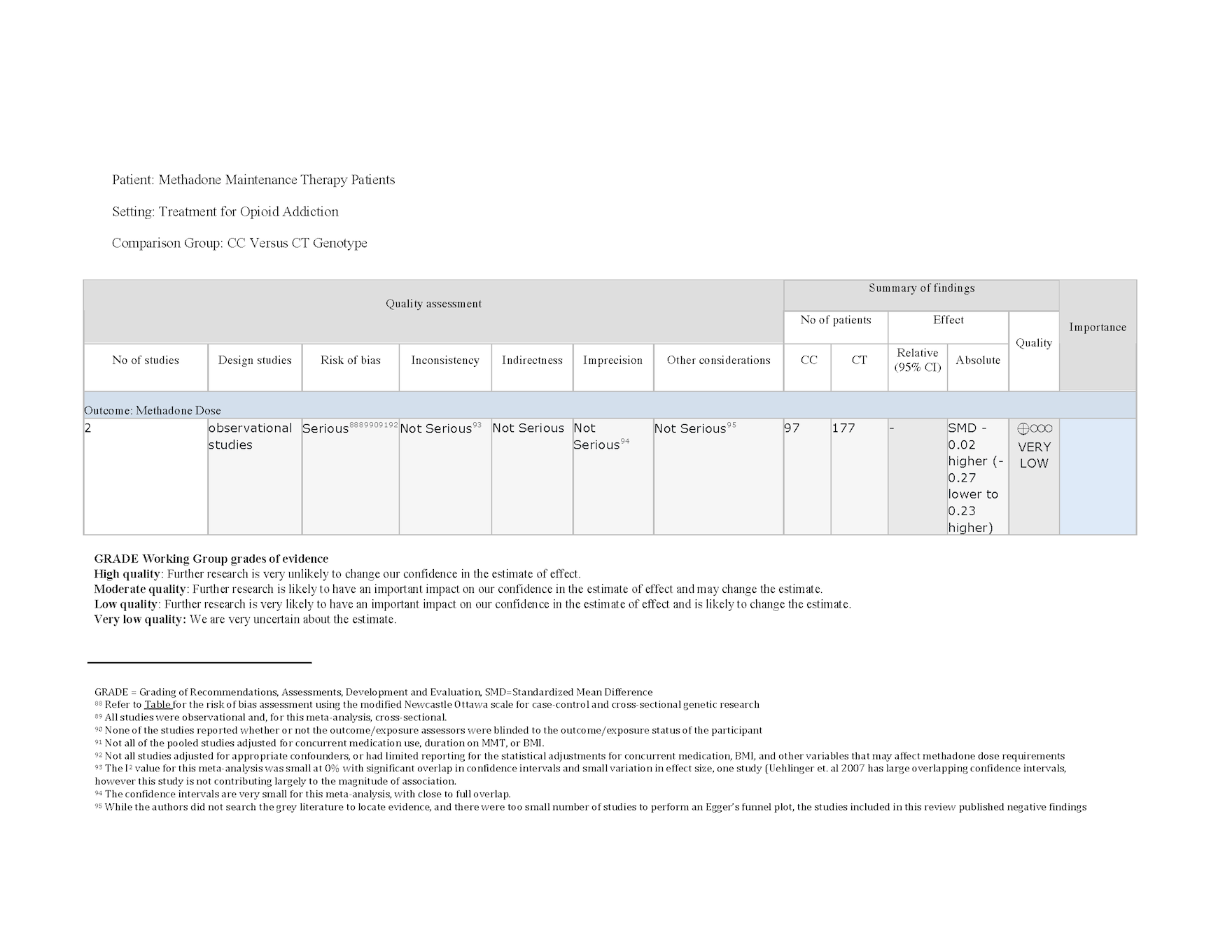

Supplement: Figure S23 — GRADE ABCB1 Methadone Dose Requirements by Genotype (CC vs CT). (TIFF) [file pone.0086114.s024.tfif]

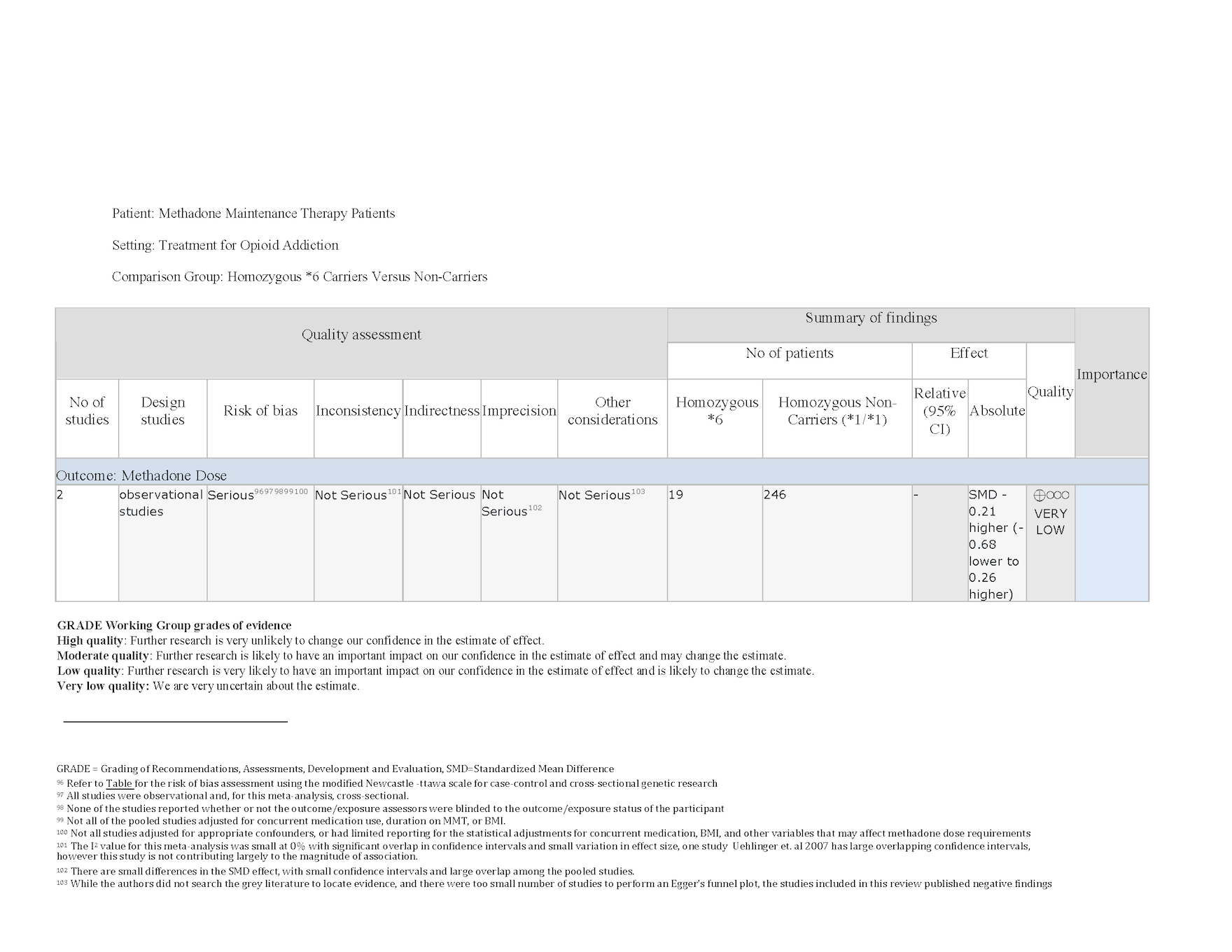

Supplement: Figure S24 — GRADE CYP2B6 Methadone Dose Requirements by *6 Genotype. (TIFF) [file pone.0086114.s025.tiff]
