# Supplementary material for: Impact of ABCB1 and CYP2B6 Genetic Polymorphisms on Methadone Metabolism, Dose and Treatment Response in Patients with Opioid Addiction: A Systematic Review and Meta-Analysis
Source: PLoS One. 2014 Jan 29;9(1):e86114. doi: 10.1371/journal.pone.0086114 (PMC3906028; doi:10.1371/journal.pone.0086114)
Supplement: Table S1 — The Genetic Determinants for MMT Response. (DOCX) [file pone.0086114.s026.docx]

| Genetic Determinants of MMT | Methadone Metabolism | Methadone Dose | Methadone Response |
| --- | --- | --- | --- |
| *ABCB1*  61A>G  3435C>T  2677G>T | [17]  [17,20,30,33]  [17] | [17]  [17,20,21,30,33]  [17,21] | [17]  [17]  [17] |
| CYP2D6 | [8,17,20] | [8,17,20] | [17] |
| CYP2B6 | [17,18,20,22,24] | [17,20,22] | [17,18] |
