# Supplementary material for: Impact of ABCB1 and CYP2B6 Genetic Polymorphisms on Methadone Metabolism, Dose and Treatment Response in Patients with Opioid Addiction: A Systematic Review and Meta-Analysis
Source: PLoS One. 2014 Jan 29;9(1):e86114. doi: 10.1371/journal.pone.0086114 (PMC3906028; doi:10.1371/journal.pone.0086114)
Supplement: Table S2 — Outline of PICO Questions for Individual SNPs and Outcomes. (DOCX) [file pone.0086114.s027.docx]

| **Population:** Patients in MMT for the treatment of opioid addiction  **Exposure:** Carriers of the Risk Allele (T) from the *ABCB1* ([rs1045642](http://snpedia.com/index.php/Rs1045642)) SNP and carriers of the *6 haplotype for the *CYP2B6* (*9 rs3745274, *4 rs2279343) Single Nucleotide Polymorphisms  **Comparison Group:** Non-Carriers of the Risk Allele (T), thus, individuals with the CC genotype for *ABCB1* ([rs1045642](http://snpedia.com/index.php/Rs1045642)) and non carriers of the *6 haplotype for the CYP2B6 gene  **Outcome:** Trough Methadone (R) and (S) enantiomer plasma levels (ng/ml)  **Question:** Among patients on MMT for the treatment of opioid addiction, do carriers of the *ABCB1* ([rs1045642](http://snpedia.com/index.php/Rs1045642)) risk allele (T) and *CYP2B6* (*9 rs3745274, *4 rs2279343) *6 haplotype have different trough methadone (R) and (S) enantiomer plasma levels (ng/ml) than patients who do not carry these variants? |
| --- |
|  |
| **Population:** Patients in MMT for the treatment of opioid addiction  **Exposure:** Carriers of the Risk Allele (T) from the *ABCB1* ([rs1045642](http://snpedia.com/index.php/Rs1045642)) SNP and carriers of the *6 haplotype for the *CYP2B6* (*9 rs3745274, *4 rs2279343) Single Nucleotide Polymorphisms  **Comparison Group:** Non-Carriers of the Risk Allele (T), thus, individuals with the CC genotype for *ABCB1* ([rs1045642](http://snpedia.com/index.php/Rs1045642)) and non carriers of the *6 haplotype for the CYP2B6 gene  **Outcome:** Methadone dose (mg/d)  **Question:** Among patients on MMT for the treatment of opioid addiction, do carriers of the *ABCB1* ([rs1045642](http://snpedia.com/index.php/Rs1045642)) risk allele (T) and *CYP2B6* (*9 rs3745274, *4 rs2279343) *6 haplotype have significantly different methadone doses (mg/d) than patients who do not carry these variants? Does the (T) allelic variant in the *ABCB1* ([rs1045642](http://snpedia.com/index.php/Rs1045642)) genotype or the *6 haplotype in the *CYP2B6* (*9 rs3745274, *4 rs2279343) SNPs affect methadone dosing among MMT patients in treatment for opioid addiction? |
|  |
| **Population:** Patients in MMT for the treatment of opioid addiction  **Exposure:** Carriers of the Risk Allele (T) from the *ABCB1* ([rs1045642](http://snpedia.com/index.php/Rs1045642)) SNP and carriers of the *6 haplotype for the *CYP2B6* (*9 rs3745274, *4 rs2279343) Single Nucleotide Polymorphisms  **Comparison Group:** Non-Carriers of the Risk Allele (T), thus, individuals with the CC genotype for *ABCB1* ([rs1045642](http://snpedia.com/index.php/Rs1045642)) and non carriers of the *6 haplotype for the CYP2B6 gene  **Outcome:** Continued opioid abuse  **Question:** Among patients on MMT for the treatment of opioid addiction, do carriers of the *ABCB1* ([rs1045642](http://snpedia.com/index.php/Rs1045642)) risk allele (T) and *CYP2B6* (*9 rs3745274, *4 rs2279343) *6 haplotype have different rates of continued opioid abuse than patients who do not carry these variants? |

**^*PICO stands for population, intervention, comparison group and outcome.^**
