# Supplementary material for: Impact of ABCB1 and CYP2B6 Genetic Polymorphisms on Methadone Metabolism, Dose and Treatment Response in Patients with Opioid Addiction: A Systematic Review and Meta-Analysis
Source: PLoS One. 2014 Jan 29;9(1):e86114. doi: 10.1371/journal.pone.0086114 (PMC3906028; doi:10.1371/journal.pone.0086114)
Supplement: Table S3 — Full Search Strategy for Systematic Review and Meta-Analysis on the Genetic Determinants of Methadone Maintenance Therapy Response. (DOCX) [file pone.0086114.s028.docx]

| CINAHL Search Strategy  Search = 1 | 1. (MH “methadone+”) or (MH “substitute opioid therapy+”) 2. (MH “disorder, substance abuse”) or (MH “substance withdrawal syndrome+”) 3. (TX “genetic variant”)  or (TX “single nucleotide polymorphism”)  or (TX “genetic polymorphism) 4. (TX “CYP-450”)  or (TX “CYP2B6”) or (TX “*ABCB1*”) or (TX “*6 (*9 rs3745274 *4 rs2279343)”) or (TX “rs1045642”) or (TX “MDR1) 5. (MH “Pharmacogentics”) 6. S1 or S2 7. S3 or S4 or S5 8. S6 and S7 |
| --- | --- |
| MEDLINE Search Strategy  Search = 54 | 1. methadone/bl, me, pk, th [Blood, Metabolism, Pharmacokinetics, Therapy] 2. limit 1 to humans 3. methadone .mp. 4. opioid substitution treatment.mp. or Opiate Substitution Treatment/ 5. limit 4 to humans 6. substance-Related Disorders/bl, dt, ge, me [Blood, Drug Therapy, Genetics, metabolism] 7. Limit 6 to humans 8. genetic polymorphism.mp. or Polymorphism, Genetic/ 9. limit 8 to humans 10. single nucleotide polymorphism.mp. or Polymorphism, Single Nucleotide/ 11. limit 10 to humans 12. polymorphism, Genetic/ or Polymorphism, Single Nucleotide/ or Genetic Variation/ or genetic variant.mp. or Phenotype/ 13. limit 12 to humans 14. Genes, MDR/ or Polymorphism, Genetic/ or *ABCB1*.mp. or Polymorphism, Single Nucleotide/ 15. limit 14 to humans 16. cytochrome P-450 Enzyme System/ or CYP*.mp. 17. limit 16 to humans 18. methadone .tw. 19. limit 18 to humans 20. 2 OR 5 OR 7 21. 3 AND 20 22. 9 OR 11 OR 13 23. 15 OR 17 24. 22 AND 23 25. 18 AND 24 |
| Web of Science Search Strategy  Search = 29 | 1. Topic=(methadone) 2. Topic=(methadone maintenance therapy) 3. Topic=(opioid substitution therapy) 4. #1 OR #2 OR #3 5. Topic=(substance withdrawal syndrome) 6. Topic=(opioid addiction) 7. Topic=(substance abuse disorder) 8. #5 OR #6 OR #7 9. Topic=(genetic variant) 10. Topic=(genetic polymorphism) 11. Topic=(single nucleotide polymorphism) 12. #9 OR #10 OR #11 13. Topic=(*ABCB1*) 14. Topic=(CYP2B6) 15. Topic=(CYP-450) 16. Topic =(MDR1) 17. Topic=(rs1045642) 18. Topic=(*9 rs3745274, *4 rs2279343) 19. #13 OR #14 OR #15 OR #16 OR #17 20. #12 OR #19 21. #4 AND #8 AND # 20 |
| Embase Search Strategy  Search = 155 | 1. methadone treatment/ or methadone.mp. or methadone/ 2. limit 1 to human 3. opioid substitution treatment.mp. or opiate substitution treatment/ 4. limit 3 to human 5. genetic polymorphism.mp. or genetic polymorphism/ 6. limit 5 to human 7. single nucleotide polymorphism.mp. or DNA/ or single nucleotide polymorphism/ or genetic variability/ or genetic polymorphism/ 8. limit 7 to human 9. gene/ or haplotype/ or *ABCB1*.mp. or single nucleotide polymorphism/ 10. limit 9 to human 11. MDR1 mp. 12. limit 11 to human 13. cytochrome P450/ or CYP-450.mp. 14. CYP2B6.mp. or cytochrome P450 2B6/ 15. limit 14 to human 16. 2 or 4 17. 6 or 8 18. 10 or 12 or 13 or 15 19. 16 and 17 and 18 |
| PsychINFO Search Strategy  Search = 15 | 1. exp Methadone Maintenance/ or exp Methadone/ or methadone.mp. 2. limit 1 to human 3. exp Treatment Outcomes/ or exp Drug Therapy/ or exp Methadone Maintenance/ or exp Opiates/ or exp Maintenance Therapy/ or opioid substitution treatment.mp. or exp Heroin/ 4. limit 3 to human 5. exp Drug Abuse/ or exp Drug Therapy/ or exp Drug Dependency/ or substance related disorders.mp. 6. limit 5 to human 7. exp Genetics/ or exp Polymorphism/ or exp Genes/ or genetic polymorphism.mp. 8. exp Polymorphism/ or exp Genes/ or single nucleotide polymorphism.mp. or exp Gene Expression/ 9. limit 8 to humans 10. exp Genes/ or exp Drug Therapy/ or exp Polymorphism/ or *ABCB1*.mp. or exp Blood/ 11. limit 10 to humans 12. exp Genes/ or exp Polymorphism/ or MDR1.mp. 13. limit 12 to humans 14. exp Genes/ or exp Polymorphism/ or rs1045642.mp. 15. limit 14 to humans 16. exp Polymorphism/ or exp Drug Therapy/ or exp Genes/ or CYP*.mp. or exp Genotypes/ 17. limit 16 to humans 18. lCYP-450.mp. 19. limit 18 to humans 20. exp Genotypes/ or exp Gene Expression/ or exp Methadone/ or exp Phenotypes/ or CYP2B6.mp. or exp Metabolism/ or exp Polymorphism/ or exp Drug Therapy/ 21. 2 or 4 22. 7 or 9 23. 11 or 13 or 15 or 17 or 19 or 20 24. 21 and 22 and 23 25. methadone .tw. 26. 24 and 25 |
