# Supplementary material for: Impact of ABCB1 and CYP2B6 Genetic Polymorphisms on Methadone Metabolism, Dose and Treatment Response in Patients with Opioid Addiction: A Systematic Review and Meta-Analysis
Source: PLoS One. 2014 Jan 29;9(1):e86114. doi: 10.1371/journal.pone.0086114 (PMC3906028; doi:10.1371/journal.pone.0086114)
Supplement: Table S4 — Modified Newcastle Ottawa Scale Tool to Asses Risk of Bias in Cross-Sectional Genetic Research. (DOCX) [file pone.0086114.s029.docx]

| **Risk of Bias** | **Criterion** | **0**  **(Definitely No: High Risk of bias)** | **1**  **(Mostly No)** | **2**  **(Mostly Yes)** | **3**  **(Definitely Yes: Low Risk of Bias)** |
| --- | --- | --- | --- | --- | --- |
| Selection Bias | **Were cohorts drawn from the same population?** | Participants with and without the outcome of interest were selected from different geographic regions, ethnicities, age groups, or patients were selected at separate time points. | The study has provided no information about the populations from which participants were selected from, and the populations were similar (i.e. time, and comparable geographic location). | The study has provided limited information about the populations from which participants were selected from, and the populations were similar (i.e. time, and comparable geographic location). | The study has provided information about the populations from which participants were selected from, and the populations were similar (i.e. time, and comparable geographic location). |
|  | **Is the source population (sampling frame) representative of the cohort of interest?** | Studies where the source population cannot be defined (or enumerated), i.e. any volunteer studies using self-recruitment. | A consecutive sample or random selection from a population that is not representative of the condition under study. | A consecutive sample or random selection from a population that is not *highly* representative of the condition under study. | A consecutive sample or random selection from a population representative of the condition under study. |
| Performance Bias | **Did the study identify and adjust for any possible influence a concurrent therapy or unintended exposure might have on the results of the investigation?** | Study does not provide information on the topic. | The study reported possible concurrent interventions and exposures, but did not explore the interaction. | The study reported and explored some concurrent therapies and/or unintended exposures. | The study reported and adjusted for all possible concurrent medications or exposures that may influence the estimates of the association between the genetic profile and outcome of interest (i*.e. If interested in understanding the relationship between genotype and methadone metabolism, concurrent medications need to be assessed before measuring methadone plasma concentration).* |
| Measurement Bias | **Was the genetic analysis of high quality and the methodology of the genetic assessment explicitly detailed?** | The study provides no information on the genotyping methods. | Limited information about genotyping is provided or the study includes polymorphisms with a low yield of genetic profile results for participants enrolled (call rate ≤90%). Hardy Weinberg Equilibrium was either not assessed or the sample did not adhere to the principle. | Either low genotyping call rate (≤90%) or inclusion of polymorphisms that did not adhere to the Hardy Weinberg Equilibrium. | The study provides a detailed description of the genotyping methods, includes included polymorphisms with a with a high yield of results for participants (call rate >90%), and statistical results assuring the Hardy Weinberg Equilibrium principle has been met during the genotyping study phase. |
| Detection Bias | **Did the study use statistical analysis methods to adjust for prognostic variables across genotyped participant groups?** | The study did not provide a summary of information regarding the distribution of known confounding variables across genotype profiles, as well the study did not statistically adjust for these prognostic variables when needed. | The study did provide a summary of known confounding variables across genotype profiles, but did not statistically adjust for these prognostic variables when needed. | The study did provide a summary of known confounding variables, but adjusted for some prognostic variables when needed. | The study provided information detailing the distribution of prognostic variables across genotyped groups. In addition, if unbalanced, the study statistically adjusted for these unbalanced variables. *(i.e. If one group of participants genotyped as CC were older than participants genotyped as TT, was age statistically adjusted for when assessing the outcome of interest?).* |
|  | **Were all outcome assessors blinded to the genetic profile information of the participant?** | There was no information about blinding of outcome assessors. | Limited information about blinding was provided; the outcome assessors were not blinded. | Some outcome assessors were blinded to the genotype of the participant. | The study reported that outcome assessments were made by members of the research team that were unaware of the genotype of the participant. |
|  | **Were all genetic assessors blinded to the outcome status of the participant?** | There was no information about blinding of genotype assessors. | Limited information about blinding was provided; the genotype assessors were not blinded. | Some genetic assessors were blinded to the outcome of the participant. | The study reported that members of the genetic assessment team were unaware of the outcome status of the participant. |
|  | **Was there an objective assessment of the outcome of interest?** | No reporting on the topic. | The study relied on self-report as the primary method of discerning participants’ outcome of interest. | The study relied on a combination of self-report and objective (e.g. laboratory) measures as the method of discerning participants’ outcome of interest. | The study used objective methods to discern the outcome status of participants, i.e. laboratory measurements, medical record linkage. |
|  | **Is there little missing data?** | **≥**15% missing data | >12 to <15% missing data | 10-12% missing data | <10% missing data |

^*This table displays the information about risk of bias for individual cross-sectional genetic studies using a new modified tool to assess risk of bias for cross-sectional genetic research.
**The tool ranks individual studies on a 0-3 scale. 0 is equivalent to high risk of bias and 3 is equivalent to low risk of bias.^
