# Supplementary material for: Impact of ABCB1 and CYP2B6 Genetic Polymorphisms on Methadone Metabolism, Dose and Treatment Response in Patients with Opioid Addiction: A Systematic Review and Meta-Analysis
Source: PLoS One. 2014 Jan 29;9(1):e86114. doi: 10.1371/journal.pone.0086114 (PMC3906028; doi:10.1371/journal.pone.0086114)
Supplement: Table S5 — Risk of Bias Table for Individual Cross-Sectional Genetic Studies. (DOCX) [file pone.0086114.s030.docx]

| **Risk of Bias** | **Criterion** | **Crettol, 2005 [**[18](#_ENREF_18)] | **Crettol, 2006** [[17](#_ENREF_17)] | **Fonseca, 2011** [[20](#_ENREF_20)] | **Levran, 2008** [[21](#_ENREF_21)] | **Uehlinger, 2007** [[48](#_ENREF_48)] |
| --- | --- | --- | --- | --- | --- | --- |
| Selection Bias | Were cohorts drawn from the same population? | **2** | **2** | **3** | **3** | **3** |
|  | Is the source population (sampling frame) representative of the cohort of interest? | **3** | **3** | **3** | **3** | **2** |
| Performance Bias | Did the study identify and adjust for any possible influence a concurrent therapy or unintended exposure might have on the results of the investigation? | **3** | **3** | **2** | **1** | **3** |
|  | Was the genetic analysis of high quality and the methodology of the genetic assessment explicitly detailed? | **3** | **3** | **1** | **2** | **1** |
| Detection Bias | Did the study use statistical analysis methods to adjust for prognostic variables across genotyped participant groups? | **3** | **3** | **2** | **2** | **3** |
|  | Were all outcome assessors blinded to the genetic profile information of the participant? | **Not Reported** | **Not Reported** | **Not Reported** | **Not Reported** | **Not Reported** |
|  | Were all genetic assessors blinded to the outcome status of the participant? | **Not Reported** | **Not Reported** | **Not Reported** | **Not Reported** | **Not Reported** |
|  | Was there an objective assessment of the outcome of interest? | **3** | **3** | **2** | **3** | **3** |
|  | Is there little missing data? | **3** | **3** | **1** | **3** | **2** |

^*This table displays the information about risk of bias for individual cross-sectional genetic studies using a new modified tool to asses risk of bias for cross-sectional genetic research.
**The tool ranks individual studies on a 0-3 scale. 0 is equivalent to high risk of bias and 3 is equivalent to low risk of bias.^
