# Supplementary material for: Impact of ABCB1 and CYP2B6 Genetic Polymorphisms on Methadone Metabolism, Dose and Treatment Response in Patients with Opioid Addiction: A Systematic Review and Meta-Analysis
Source: PLoS One. 2014 Jan 29;9(1):e86114. doi: 10.1371/journal.pone.0086114 (PMC3906028; doi:10.1371/journal.pone.0086114)
Supplement: Table S6 — Risk of Bias Table using the Modified Newcastle Ottawa Scale for Genetic Case Control Studies. (DOCX) [file pone.0086114.s031.docx]

| **Risk of Bias** | **Criterion** | **Hung, 2011 (8 stars)** [[30](#_ENREF_30)] | **Coller, 2006 (4 stars)** [[33](#_ENREF_33)] |
| --- | --- | --- | --- |
| Selection Bias | Is the case definition adequate? | ⊕ | ⊗ |
|  | Was there a consecutive or obviously representative series of cases? | ⊕ | ⊕ |
|  | Were controls selected from the community? | ⊕ | ⊗ |
|  | Definition of control: Were controls disease free? | ⊕ | ⊗ |
| Detection Bias | Comparability of cases and controls on the basis of the design or analysis:  a) study controls for concurrent medication of duration of treatment | ⊕ | ⊗ |
|  | Ascertainment of exposure included an objective measurement (i.e. proper genotyping with a good call rate and in HW equilibrium) | ⊕ | ⊕ |
|  | Was there the same method of exposure ascertainment for cases and controls? | ⊗ | ⊕ |
|  | Is there little missing data? | ⊕ | ⊕ |

^*The Newcastle Ottawa scale is evaluated using a system of stars, where nine stars is the maximum rating an individual study can achieve. In this chart, the ⊕ symbol represents a star.^
